# CARF enrichment promotes epithelial–mesenchymal transition via Wnt/β-catenin signaling: its clinical relevance and potential as a therapeutic target

**DOI:** 10.1038/s41389-018-0048-4

**Published:** 2018-05-11

**Authors:** Rajkumar S. Kalra, Anupama Chaudhary, A-Rum Yoon, Priyanshu Bhargava, Amr Omar, Sukant Garg, Chae-Ok Yun, Sunil C. Kaul, Renu Wadhwa

**Affiliations:** 1 Drug Discovery and Assets Innovation Lab, DBT-AIST International Laboratory for Advanced Biomedicine (DAILAB), DAICENTER, National Institute of Advanced Industrial Science & Technology (AIST), Tsukuba, Ibaraki 305-8565 Japan; 2 Department of Bioengineering, College of Engineering, Hanyang University, Seongdong-gu, Seoul, Korea

## Abstract

CARF (Collaborator of ARF)/CDKN2AIP was discovered as a novel ARF-binding protein. It has been established as an essential cell survival, p53-, and cell proliferation-regulatory protein. Although a moderate upregulation of CARF caused growth arrest and senescence, its excessively enriched levels were shown to facilitate aggressive proliferation and malignant transformation of cancer cells. Here, we examined the relevance of CARF levels in clinical tumors and found its amplification (both at gene and transcript levels) in a variety of invasive and metastatic malignancies. Consistent with the clinical readouts, enrichment of CARF in cancer cells promoted epithelial–mesenchymal transition (EMT). Cancer database and molecular analyses revealed that it activates Wnt/β-catenin signaling axis, as evident by enhanced nuclear localization and function of β-catenin marked by increased level of SNAIL1, SNAIL2, ZEB1, and TWIST1 and its downstream gene targets. Of note, targeted knockdown of CARF led to decrease in nuclear β-catenin and its key downstream effectors, involved in EMT progression. Consistent with this, CARF targeting in vivo either by naked siRNA or CARF shRNA harboring adeno-oncolytic virus caused suppression of tumor progression and lung metastasis. Taken together, we report clinical and therapeutic relevance of CARF in EMT and cancer invasiveness/metastasis, and propose it as a potent therapeutic target of aggressive cancers.

## INTRODUCTION

Systematic diagnosis, screening, and targeted therapeutic approaches have considerably improved clinical response and survival of cancer patients in last decade. Furthermore, molecular profiling of aggressive tumors has not only led to improved prognosis, but also helped to minimize the therapeutic side effects in several common malignancies^1,2^. However, risk of metastases and recurrence of disease remained largely inevitable and unpredictable, and hence warrant continued efforts to characterize the associated underlying mechanisms. Transformed cancer cells progressively loose cell–cell adhesion/epithelial traits and acquire invasive mesenchymal characteristics via a systemic cellular reprograming, viz. epithelial-to-mesenchymal transition (EMT). EMT facilitates spread of cancer from the site of origin to other organs via acquiring motility, activation of extra-cellular matrix (ECM)-degrading proteases enabling invasion and eventual dissemination through vascularization^3^. Conferring reorganization of cytoskeleton and cellular polarity, it plays indispensable role in biological processes including embryonic development and wound healing^4,5^, and has been tightly associated with cancer metastases, drug resistance, and recurrence^6–8^.

During the process of EMT, cells progressively loose set of proteins required for cell-adhesion/tight-junction (e.g., E-cadherin, occludin, and ZO-1) and gains others (e.g., vimentin, N-cadherin, or fibronectin) that offer mesenchymal properties^6,9^. Transcription factors, viz. Slug, Snail, and Twist have been demonstrated to regulate this process and are in turn influenced by diverse signaling pathways including TGF-β1, RTK (Receptor Tyrosine Kinase) receptors, i.e., EGFR, ERBB2/HER2-AKT, ITGB1/FAK, NOTCH, and Wnt/β-catenin proteins^4,10^. In particular, deregulated function of TGF-β1, a regulatory cytokine has been associated with tumor initiation and metastases^11^. It is frequently amplified in metastatic breast and pancreatic cancers^12^ and found to promote malignant and metastatic characteristics via activation of Snail and Zeb1^13^. Similarly, the RTK receptors, EGFR and ERBB2 (frequently amplified in glioblastoma, breast and esophagus cancers) have been shown to promote EMT^12^. Focal adhesion kinase (FAK) and AKT, serine/threonine kinases are upregulated and shown to promote EMT in various malignancies^12^. Upregulation of Wnt in cancer cells has been shown to stabilize β-catenin and promote its translocation to the nucleus, where it serves as a coactivator of TCF-LEF transcription factors and regulate several EMT regulating proteins including repression of E-cadherin and activation of Snail, Zeb1, and Vimentin^3,10,13^.

We have previously cloned CARF (the Collaborator of ARF), as a novel interactor of tumor suppressor protein p14^ARF^ (ARF)^14^. It has been shown that CARF activates p53, a key tumor suppressor in a ARF-dependent and independent manners^15,16^ leading to execution of growth arrest and pre-mature senescence, as marked by p21^WAF1^ activation in human cancer and normal cells, respectively^17,18^. Excessively enriched or super-high expression level of CARF was shown to promote pro-proliferation and malignant properties of cancer cells in a feedback regulatory manner involving p53-HDM2 and DNA damage regulating proteins (Chk1, Chk2, ATM, and ATR)^18^. Most recently, we found that CARF led to transcriptional repression of p21^WAF1^ and contributes to malignant transformation remarkably detected in p53-deficient cells^19^. In light of above information, in the present report, we investigated clinical relevance of CARF in tumorigenesis and its progression. An initial survey marked amplified CARF levels in invasive and metastatic malignancies in public cancer patient databases and in clinical tumor samples. We generated CARF-enriched cells and performed in vitro and in vivo assays, and found that CARF enrichment leads to EMT progression via Wnt/β-catenin signaling axis as evidently found by β-catenin nuclear translocation and activation of TCF4/β-catenin transcriptional targets. CARF knockdown, on the other hand, abolished these activities both in in vitro and in vivo models demonstrating functional relevance of CARF in EMT progression and its importance as a therapeutic target for metastatic malignancies.

## RESULTS

### CARF-amplification marks tumor invasiveness and metastases

**Fig. 1:**
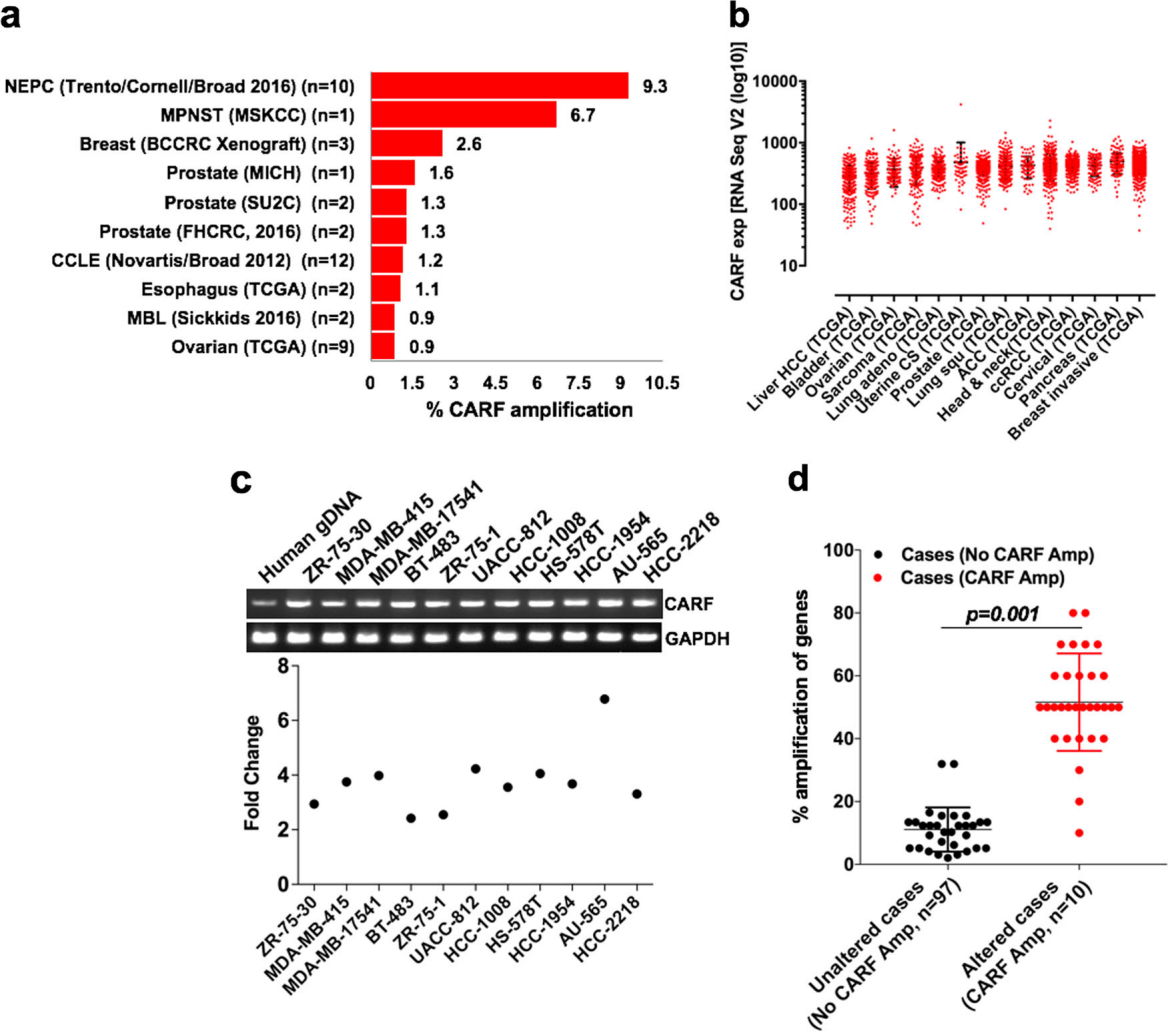
Amplification of CARF and its enrichment in metastatic malignancies. **a** Percentage CARF amplification in clinical cancer datasets in TCGA database. **b** Enrichment of CARF mRNA levels in invasive and metastatic malignancies; RNA-Seq (V2 algorithm) expressions in log10 scale. **c** CARF-gene amplification in metastatic breast cancer cell lines (upper); values represented as fold enrichment (lower). **d** Dot plot showing percentage amplification of candidate genes (shortlisted in TCGA-defined invasion/metastases and angiogenesis gene sets) in patients with (*n* = 10) and without (*n* = 97) CARF amplification in NEPC (2016) dataset

In order to examine the status of CARF across malignancies, we surveyed its genomic alterations (Copy Number Alterations, CNA) in clinical tumor patient database (TCGA). As shown in Fig. 1a, we found CARF amplification in several tumor types of which prostate cancer (Neuroendocrine; NEPC, Trento/Cornell/Broad 2016 dataset) scored the highest (9.3%). Expression data revealed elevated CARF mRNA levels across datasets in various malignant tumors (Fig. 1b; Figure S1a). We next crosschecked CARF levels at Oncomine, an online cancer microarray expression database, and found its amplification in invasive breast carcinomas as compared to normal tissue (Figure S1b). Several other invasive malignancies such as sarcoma, lung, and prostate cancers, showed enrichment of CARF expression at Oncomine, suggesting clinical relevance of CARF upregulation with metastatic cancers. In view of these findings, we examined and found genomic amplification of CARF (2–7 fold) in number of invasive breast cancer cell lines, derived from metastatic sites (Fig. 1c). In order to further confirm CARF correlation with metastasis, we examined the status of 31 genes (pooled from defined invasive/metastatic and angiogenesis gene sets, at TCGA) in NEPC (2016) dataset having patients with (*n* = 10) and without (*n* = 97) CARF amplification. As shown in Fig. 1d, we found that patients with CARF amplification showed 40% or higher amplification of 28 out of 31 genes. Three genes (MMP2, MMP15 and CDH1) showed low (<20%) level of amplification. Of note, CDH1, shown to decline during invasion or EMT progression^20–22^, showed the least change in CARF-amplified patients. These data strongly suggested that CARF amplification is associated with metastatic properties of the cancer cells.

**Fig. 2:**
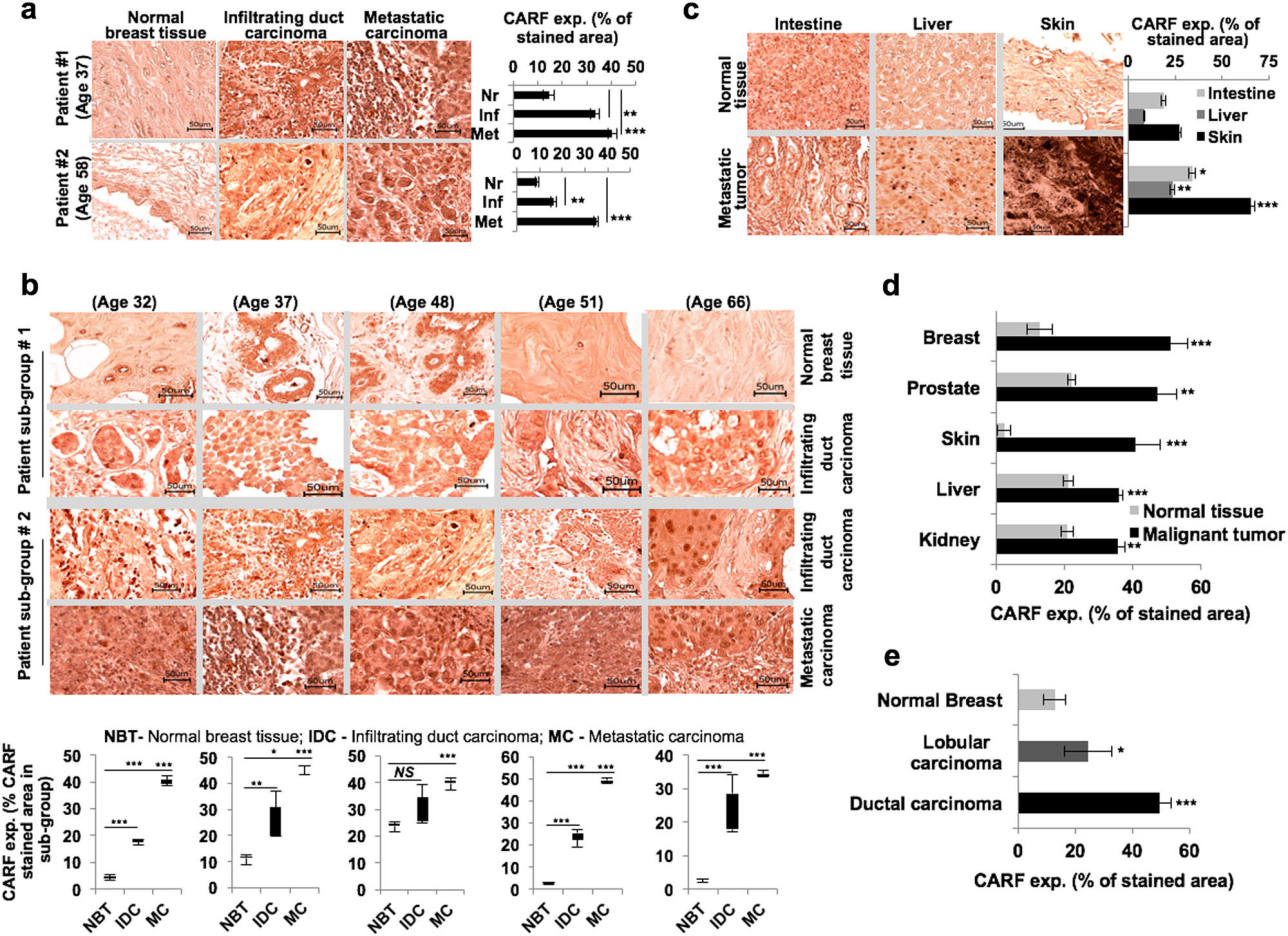
CARF enrichment correlates with metastatic progression in clinical tumors. **a** Immunohistochemical staining showing CARF expression in normal, infiltrating, and metastatic breast cancer patient tumors; their quantitative levels shown on right. **b**, CARF levels in patients with normal to infiltrating (sub-group #1) and infiltrating to metastatic (sub-group #2) breast tumors in different age groups; quantitation shown below. **c** Expression of CARF in normal and metastatic tumor sections of intestine, liver and skin; quantitation shown on right. **d** Quantitation of CARF expression in normal and malignant breast, prostate, skin, liver, and kidney tumors sections; and in normal, lobular, and ductal breast cancer immunohistological sections retrieved from Human Protein Atlas (**e**)

We next examined the expression level of CARF in clinical tumor sections using tissue microarray. Of note, a significant and gradual increase in CARF was detected from progression of infiltrating to metastatic breast carcinoma as compared to the basal level in normal matched tissues (Fig. 2a). Comparison of two patient sub-groups of similar age, bearing normal, infiltrating and metastatic tumors, revealed significant CARF enrichment in the latter stages (Fig. 2b). While a moderate increase in CARF expression was detected in intestine and liver malignant tumors as compared to their matched normal tissues, metastatic melanoma showed abundant enrichment as compared to normal skin (Fig. 2c). We crosschecked it with the Human Protein Atlas and observed enriched CARF expression in breast, prostate, skin, liver and kidney carcinomas as compared to their respective normal tissues (Fig. 2d). Consistent to mRNA expression data (Figure S1b), invasive ductal tumors showed a higher level of CARF expression than the lobular breast carcinoma (Fig. 2e). Together with the data in Fig. 1, these findings confirmed the involvement of CARF in cancer metastasis.

### CARF enrichment enhanced cancer cell migration and invasiveness

**Fig. 3:**
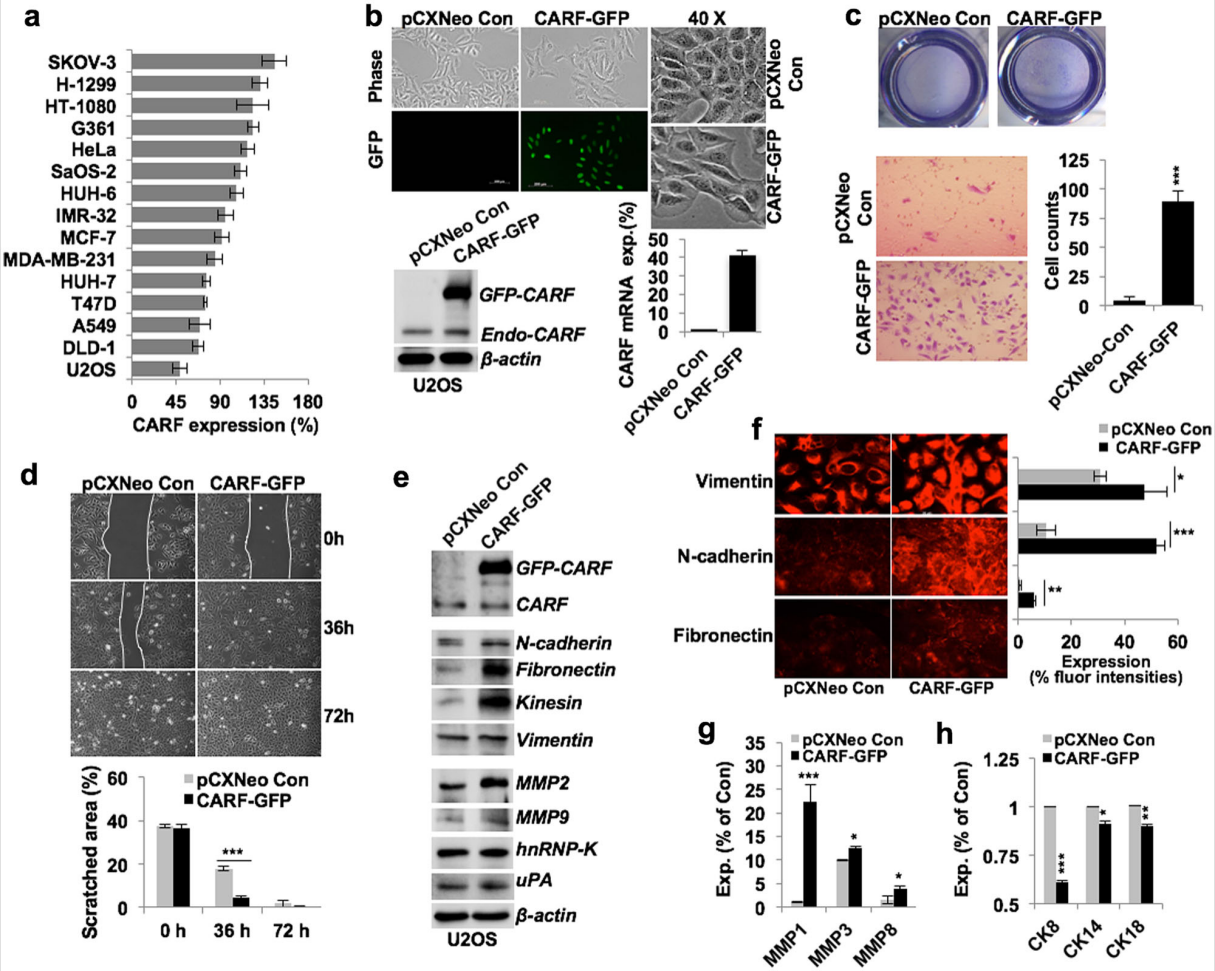
CARF enrichment enhanced cancer cells migration and invasiveness. **a** Quantitation showing comparative protein expression of CARF in cancer cell lines. **b** Cell morphology of U2OS cells infected with control/pCXNeo vector and CARF-GFP retro-virus, shown at ×10 and ×40 magnifications. Immunoblot and qPCR data showing CARF protein and transcript expression stable cells is shown below. **c** Matrigel invasion assay showing invaded cells (wells, at top) and count (inset images, below) of control and CARF-GFP cells; quantitation shown on the right. **d** Wound-healing assays (72 h) showing comparative migration of control and CARF-enriched cells, quantitation shown below. **e** Immunoblots showing increased expressions of mesenchymal (n-cadherin, fibronectin, kinesin, and vimentin) and invasive (MMP2, MMP9, hnRNP-K, and uPA) markers in CARF-GFP cells. **f** Immunostaining of vimentin, N-cadherin, and fibronectin showing increase in their expression in CARF-GFP cells, quantitation of their fluorescent intensities is shown on right. Quantitation of mRNA levels showing increased invasive (**g**; MMP1, MMP3, and MMP8) and decreased epithelial (**h**; CK8, CK14, and CK18) markers, respectively, in CARF-GFP cells, as compared of control cells

In order to get molecular insights to the role of CARF in EMT and cancer metastasis, we recruited a panel of in vitro cultured cancer cells and examined their level of CARF expression. We found that invasive ovarian (SKOV-3), lung (H-1299), and fibrosarcoma (HT-1080) cell lines derived from secondary metastatic tumor site possessed high level of CARF expression (Fig. 3a). Although the non-metastatic osteosarcoma (U2OS) showed lowest level of CARF expression (Fig. 3a). Analyses of CARF status in cancer cell lines in CCLE (Novartis/Broad 2012) TCGA dataset also revealed CARF-gene amplification and transcript enrichment in number of circulating/blood-born and invasive cancer cell lines derived either from primary or secondary metastatic tumor site (Figure S1c) suggesting that CARF may have a key role in cancer metastasis.

We next generated CARF-enriched U2OS and SKOV-3 cells, representing low and high endogenous CARF levels, respectively. To distinguish it from endogenous CARF, the ectopic CARF was tagged with GFP (Fig. 3b). Cell phenotype analyses revealed that CARF enrichment enhanced their mesenchymal characteristics (Fig. 3b). As shown in Figs. 3c and d, the CARF-enriched cells exhibited higher migration and invasiveness. Analysis of several EMT marker proteins in control and CARF-GFP U2OS and SKOV-3 cells revealed their upregulation in the latter (Fig. 3e; Figure S2a). Consistently, enhanced expression of vimentin, N-cadherin, and fibronectin in U2OS/SKOV-3 CARF-enriched cells confirmed promotion of their mesenchymal characteristics (Fig. 3f; Figure S2b). It was further affirmed by upregulated MMP1, MMP3 and MMP8, and hnRNP-K (Fig. 3g; Figure S2c) and downregulated cytokeratins levels in these cells (Fig. 3h).

### CARF enrichment promoted EMT via activating Wnt/β-catenin signaling

Considering the above data that demonstrated the role of CARF enrichment in EMT, we next investigated enrollment of key EMT regulating signaling axes. Canonical factors including TGF-β, tyrosine kinase receptors (i.e., EGFR, HER2/ErbB2), Wnt/β-catenin, and NOTCH1 have been established as the key regulators of EMT^6,23,24^. We next examined their correlation with CARF in TCGA cancer database. In a co-occurrence-based analyses, taking amplification of these key modulators in their top 10 datasets, we found an association of β-catenin amplification with CARF (Figure S3a). As no consistent association of any of the other regulators, TGFB1, EGFR, ERBB2 and NOTCH1 was found with CARF amplification (Figure S3b–e). To test it further, we analyzed status of transcriptional targets of TCF4/β-catenin in NEPC (2016) dataset. A large proportion of these genes were found to be significantly upregulated in above dataset (Figure S3f). Of note, TCF4/β-catenin direct target genes involved in regulating EMT process showed a significant increase, except of CDH1 i.e. a repressive target (Figure S4). Furthermore, dataset exhibited a positive association in the expression level of indirect TCF4/β-catenin gene targets categorized as repressor and activators of EMT process (Figure S4). Gene amplification frequencies in the dataset also revealed an association of CARF with that of DVL2, TCF4, LEF1, and JUN genes suggested to make a complex to stabilize TCF4/β-catenin interaction in the nucleus (Figure S5a). Besides these, amplification of number of β-catenin gene targets including TDG, SIAH1, CSNK1A1, BTRC, FRAT1, MAP3K7, SMAD4, and TLE1 also found to be associated with CARF (Figure S5b). These data suggested that CARF might be involved in regulation of Wnt/β-catenin signaling.

**Fig. 4:**
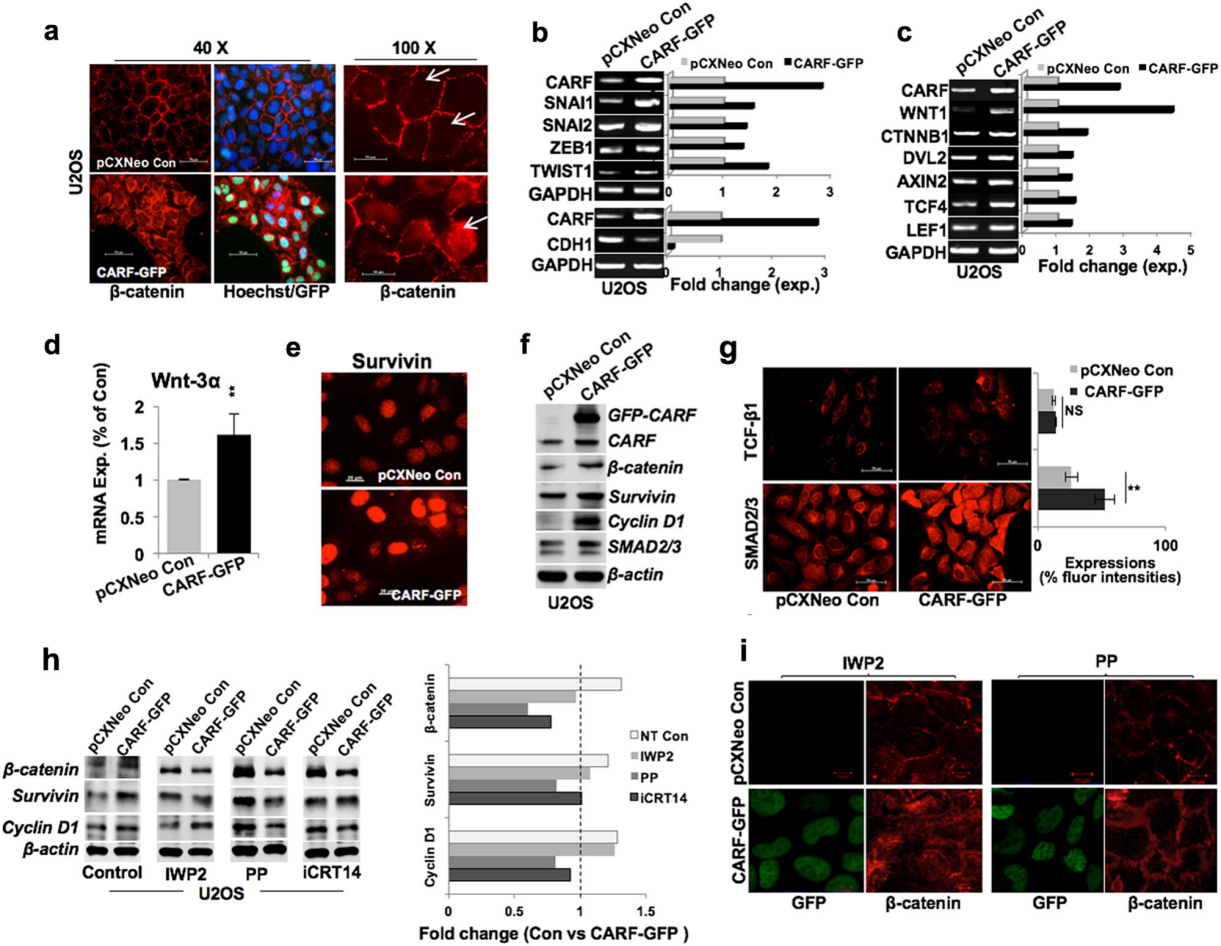
**CARF enrichment promoted EMT via activating Wnt/β-catenin signaling.** **a** Immunostaining showing β-catenin expression in control and CARF-enriched cells; nuclear enrichment of β-catenin is clearly seen in the latter in image on the right (×100, marked by arrow). **b** mRNA expression of transcription factors (SNAIL1, SNAIL2, ZEB1, TWIST1, and CDH1) in control and CARF-enriched cells. **c** Transcript levels of β-catenin target genes (WNT1, CTNBB1, DVL2, AXIN2, TCF4, and LEF1); quantitation (fold change) is shown on the right. **d** qPCR-based mRNA quantitation showing increased Wnt3α in CARF-enriched cells. **e** Immunostaining showing survivin expression. **f** Immunoblots showing increase in β-catenin and its target proteins viz. survivin, cyclin D1, and SMAD2/3 in CARF-GFP cells. **g** TCF-β1 and SMAD2/3 immunostaining showing an increase in latter, quantitation shown on right. **h** Immunoblots showing protein levels of β-catenin and its targets (Survivin, Cyclin D1) in control, IWP2-, PP- and iCRT14-treated control (U2OS pCXNeo), and CARF-enriched (CARF-GFP) cells, quantitation is shown on right. **i** β-catenin immunofluorescence in IWP2- and PP-treated cells; lack of nuclear β-catenin was observed in PP-treated cells

To investigate the above correlation in vitro, we examined β-catenin expression in U2OS/CARF-GFP cells and found its remarkable enrichment in the nucleus (Fig. 4a), suggesting its transcriptional activation. The latter was endorsed by upregulation of β-catenin gene targets, SNAI1, SNAI2, ZEB1, and TWIST1 transcription factors, known to be the key instigators of EMT process (Fig. 4b; Figure S6a). Of note, SNAI1 and TWIST1, two direct targets of β-catenin exhibited greater increase as compared to the SNAI2 and ZEB1, i.e., the indirect targets of β-catenin, in both U2OS and SKOV-3/CARF-GFP cells (Fig. 4b, Figure S6a). Of note, we observed a repression of CDH1 transcript levels in these cells (Fig. 4b, Figure S6a). Transcript levels of a number of gene targets that interact in cytoplasm, e.g., DVL2, AXIN2, and nucleus, e.g., TCF4, LEF1 to form TCF4/β–catenin complex were upregulated in CARF-enriched U2OS (Fig. 4c) and SKOV-3 cells (Figure S6b). Together, these data suggested that CARF enrichment led to activation of TCF4/β-catenin function and repression of E-cadherin, an epithelial marker; declines during EMT progression. Furthermore, steady expression of TGFβ1, its receptors (i.e., TGFBR1) and of FAK and AKT, the key EMT regulators of TGFβ and Tyrosine/Focal-adhesion-kinase signaling axis, respectively, further ruled out their involvement in CARF-promoted EMT function in these cells (Figures S6c–d). On the other hand, CARF-GFP cells showed an increase in expression of Wnt ligands viz. Wnt1 (Fig. 4c; Figure S6b) and Wnt3α (Fig. 4d).

Consistent to the increase in β-catenin transcript, and its nuclear translocation (Fig. 4a), we found increased expression of nuclear survivin in CARF-enriched cells (Figure 4e). β-catenin, Survivin, SMAD2/3, and Cyclin D1 proteins also showed an increase in CARF-enriched U2OS and SKOV-3 cells (Fig. 4f, Figure S6e). Of note, TGFβ1 protein levels remained unchanged in these cells (Fig. 4g), consistent to its transcript levels (Figure S6c). On the other hand, upregulation and nuclear translocation of SMAD2/3, also a gene target of β-catenin further implies its regulation in CARF-enriched cells (Fig. 4g). These data suggested that CARF regulates nuclear translocation of β-catenin, thereby instigating its transcriptional function. To investigate further the mechanism of CARF-driven β-catenin activation, we considered two possibilities; involvement of (i) Wnt ligand and (ii) kinase pathway. Accordingly, we enrolled IWP2, a specific inhibitor of Wnt ligand^25^ and Pyrvinium pamoate (PP), an allosteric activator of Casein Kinase 1α (CK1α)^26^ that stimulates ubiquitination and degradation of β-catenin by its phosphorylation^27^. As shown in Fig. 4h, in contrast to the untreated CARF-enriched control U2OS cells, PP-treated cells showed marked decrease in β-catenin protein and its targets (Survivin and Cyclin D1); such decrease was even more evident in CARF-enriched SKOV-3 cells (Figure S7a). Similar results were obtained with iCRT14, a potent inhibitor that interrupts β-catenin and TCF4 binding and halts β-catenin-mediated transcription (Fig. 4h, Figure S7b). On the other hand, treatment of IWP2 appeared not to significantly alter CARF-induced upregulation of β-catenin, Survivin and Cyclin D1 (Fig. 4h). These data suggested that CARF-driven activation of β-catenin function is essentially regulated through kinase pathway. These results were further supported by immunostaining of β-catenin. As shown in Fig. 4a and i, CARF-enriched control and IWP2, and not the PP, treated cells showed nuclear translocation of β-catenin. Similar results were obtained in SKOV-3 cells (Figure S7c).

### CARF knockdown attenuated cancer cell invasion and migration via abrogating nuclear translocation of β-catenin

**Fig. 5:**
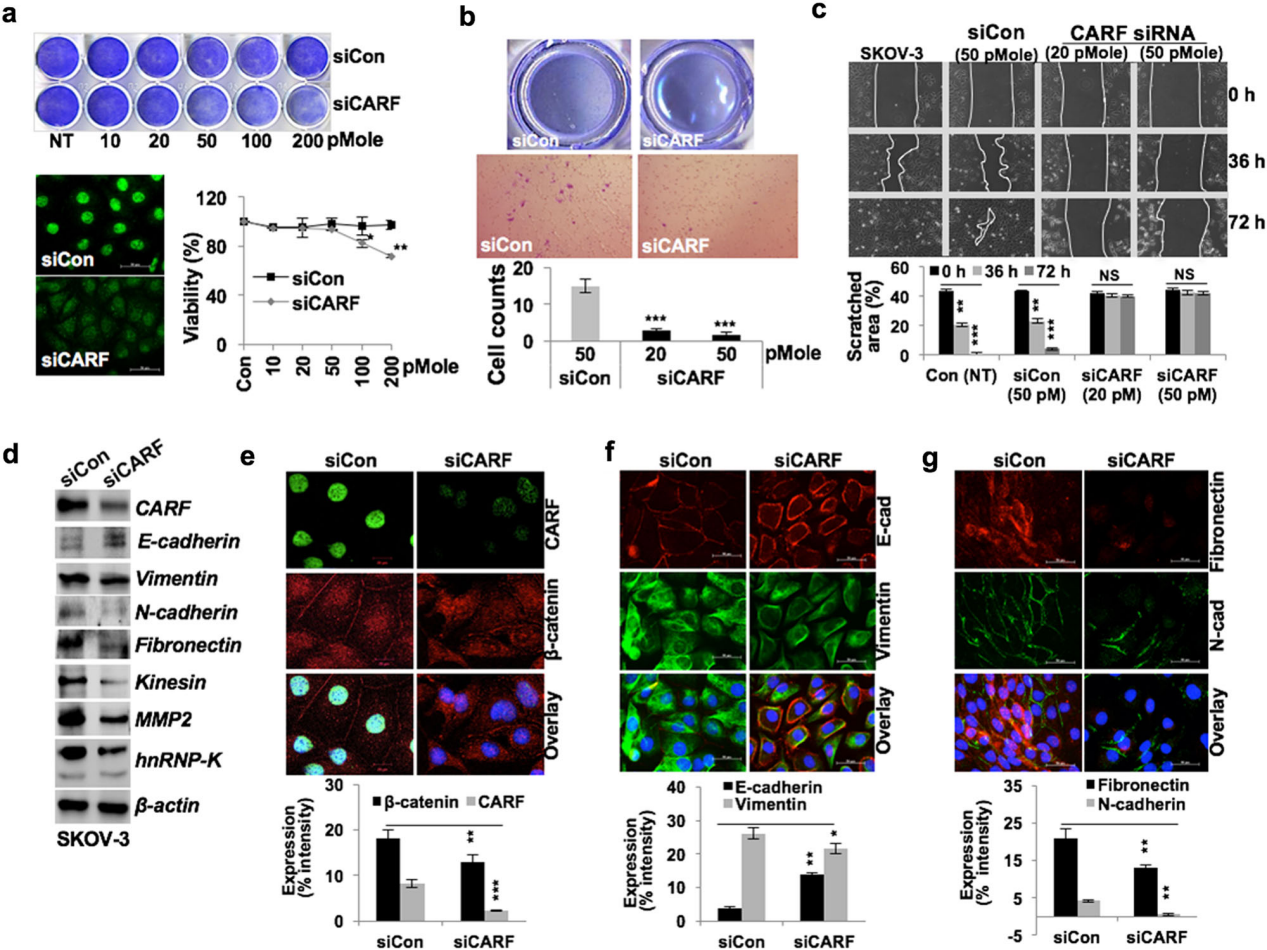
CARF knockdown attenuated cancer cell invasiveness and abolished EMT phenotype. **a** Control and CARF siRNA transfected crystal violet-stained SKOV-3 cells showing reduced proliferation in latter with increasing siRNA (10–200 pM) concentrations. Immunostaining of CARF (with 50 pM siRNA) and cell viability (10–200 pM), respectively. **b** Invasion assay showing invaded cells and quantitation (below) in control and CARF knockdown cells. **c** Wound-healing assay (72 h) showing reduced cell migration in CARF knockdown cells; quantitation shown below. **d** Immunoblotting and **e–g** immunostaining of CARF, β-catenin (**e**), E-cadherin and vimentin (**f**), and fibronectin and N-cadherin (**g**), showing decrease in CARF knockdown cells; E-cadherin showed increase; quantitation of their fluorescence intensities are shown below

We next investigated whether acquired EMT characteristics in CARF-enriched cells via activation of Wnt/β-catenin axis could be reversed by CARF suppression. CARF-specific siRNA (20–200 pM; 72 h) mediated suppression of CARF in SKOV-3 cells (higher level of endogenous CARF expression) resulted in reduced survival, a housekeeping CARF function (Fig. 5a) as also reported earlier^28^. We opted to use sub-toxic concentrations (20–50 pM) of CARF siRNA for 48 h in in vitro assay. CARF-compromised SKOV-3 cells showed decrease in migration and invasion properties (Figs. 5b, c). Furthermore, dose-dependent inhibition of CARF siRNA (~10–50 pM, sub-toxic doses) on migration was observed in highly invasive cancer cells (viz*.* HT-1080 and MDA-MB-231) (Figures S8a and S8b). These cells led to a gradual inhibition of cell migration with increasing CARF siRNA concentration, as observed by cell morphology and activity in wound-healing assay (Figures S8c–S8d and data not shown). Analysis of protein expressions revealed downregulation of number of mesenchymal and invasion marker (Fig. 5d), while E-cadherin levels elevated. Of note, compared to control, CARF-compromised SKOV-3 cells showed a dispersed and reduced nuclear β-catenin accumulation (Fig. 5e). Additionally, CARF-silenced cells possessed higher E-cadherin and reduced vimentin (Fig. 5f), N-cadherin, and fibronectin (Fig. 5g) expression as compared to the control cells.

**Fig. 6:**
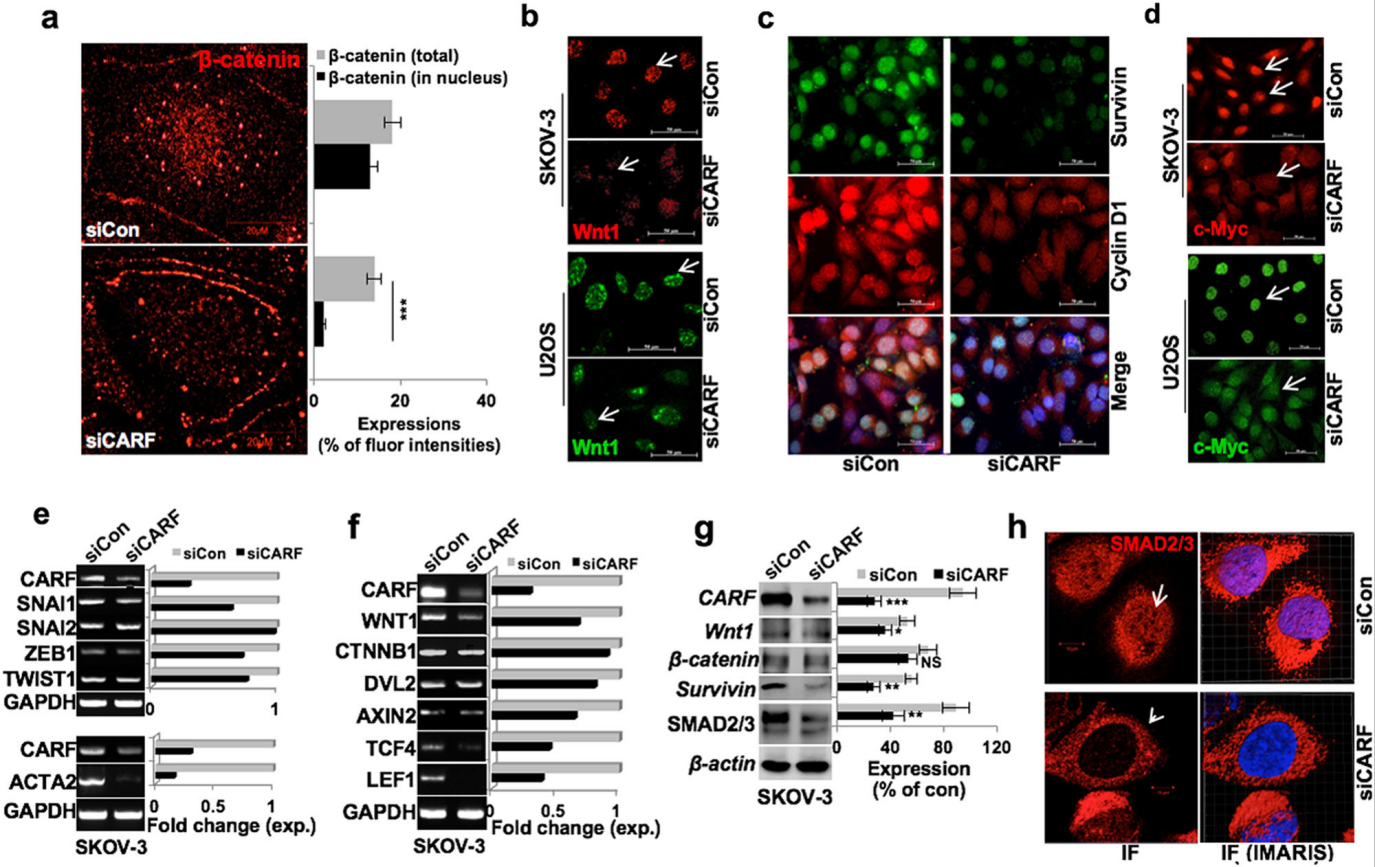
CARF knockdown diminished EMT progression via abrogating β-catenin nuclear translocation. **a** Immunostaining showing depletion of β-catenin expression in nucleus in CARF knockdown cells; quantitation of total and its nuclear level is shown at right. **b** Wnt1 immunostaining showing reduced nuclear foci in both, SKOV-3 and U2OS CARF-compromised cells. **c** Survivin and Cyclin D1 immunostaining showing their reduced levels in SKOV-3 CARF/siRNA cells. **d** c-Myc immunostaining showing a decrease in its expression in CARF knockdown both, SKOV-3 and U2OS cells. **e** Transcript levels of the SNAIL1, SNAIL2, ZEB1, TWIST1, and ACTA2 (αSMA), and **f** the β-catenin target genes; WNT1, CTNBB1, DVL2, AXIN2, TCF4, and LEF1 showed decrease in CARF knockdown cells; quantitation in fold change is shown on the right. **g** Immunoblots showing decrease in β-catenin and its effector proteins viz. Wnt1, survivin, and SMAD2/3 in SKOV-3 siCARF cells. **h** Immunostaining showing reduced SMAD2/3 nuclear localization in SKOV-3 siCARF cells; interactive IMARIS image with semi-transparent nuclear (blue) are shown on the right

We next determined the role of Wnt/β-catenin in CARF-induced EMT. As shown in Fig. 6a, CARF silencing led to a sixfold decrease in nuclear β-catenin expression; although the total β-catenin level did not show significant change (Fig. 6a). CARF silencing also led to decrease in Wnt1 nuclear foci in both U2OS and SKOV-3 cells (Fig. 6b). Of note, nuclear levels of survivin and cyclin D1 showed decrease (Fig. 6c). Similarly, these cells showed a reduced c-Myc accumulation in nucleus in both U2OS and SKOV-3 cells (Fig. 6d). We found that CARF-compromised cells possess reduced level of SNAI1 and TWIST1, the direct targets of β-catenin (Fig. 6e). Although SNAI2 and ZEB1 transcript levels altered slightly. These data suggested that CARF silencing led to abrogation of TCF4/β-catenin function marked greater effect on its immediate/direct targets (SNAI2, ZEB1). α-Smooth Muscle Actin (α-SMA/ACTA2), a key EMT marker also showed significant decrease in CARF-compromised cells (Fig. 6e, lower). Analysis of transcript levels of Wnt1 and β-catenin partners in cytoplasm and nuclear complex revealed that along with Wnt1, AXIN2, TCF4, and LEF1 showed a significant decrease, although DVL2 decrease marginally (Fig. 6f). Of note, β-catenin transcript level remained largely unchanged in control and CARF-compromised cells (Fig. 6f). On the other hand, the latter showed nuclear depletion of β-catenin, with its unaltered total protein levels (Figs. 6a, g) suggesting an inhibition of its transcriptional activation function by its nuclear depletion as was endorsed by reduced expression of its gene targets and effector proteins. As shown in Fig. 6g, Wnt1, Survivin, and SMAD2/3 were reduced in CARF-compromised cells. Besides these, we observed nuclear depletion of SMAD2/3 in CARF-compromised cells (Fig. 6h). The results were confirmed in CARF-compromised U2OS cells (Figure S9a–c). Of note, these cells, as in SKOV-3, showed SMAD2/3 depletion from the nucleus (Figure S9d), suggesting an inhibition of its nuclear function, downstream of TCF4/β-catenin activity, critically involved in EMT.

### CARF knockdown attenuated tumor metastases in xenograft models

**Fig. 7:**
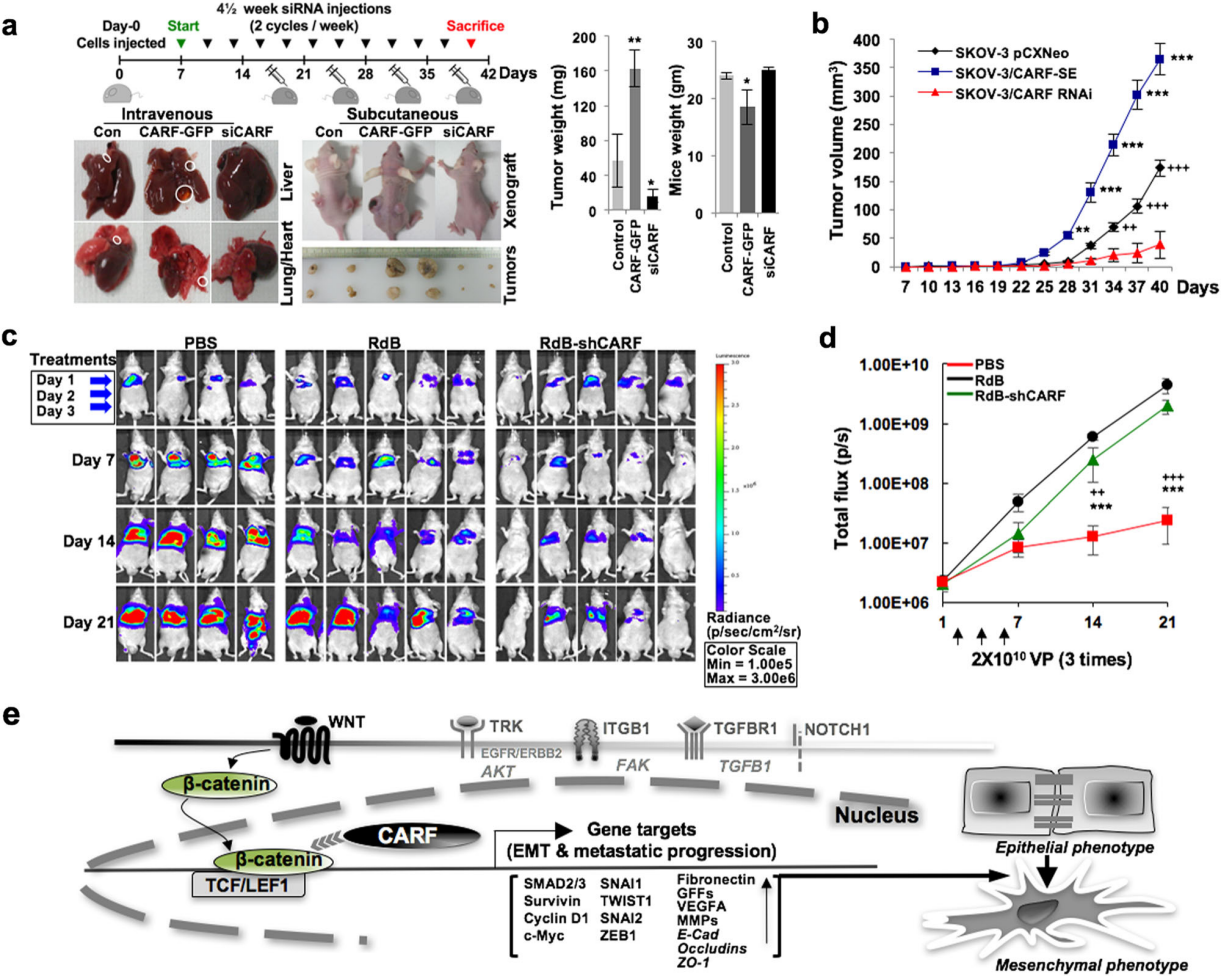
CARF knockdown attenuates tumor metastases in xenograft models. **a** Schematic diagram showing regime of nude mice xenograft assays. **b** Tumor volumes of control, CARF-enriched, and CARF-compromised xenografts. **c**, Luciferase activity of orthotopic xenograft tumors in mice systemically treated with PBS, RdB, or RdB-shCARF; luciferase intensity and its scale bar are shown on the right. **d** Quantitation plot showing total flux (p/s) values (tumor size) retrieved from PBS, RdB, or RdB-shCARF groups on 7, 14, and 21 days. **e** Schematic model showing role of CARF in EMT progression, it stimulates nuclear localization of β-catenin instigating activation of several key transcription factors and gene targets that promote progression of EMT

To examine the physiological relevance of CARF-promoted EMT and its abrogation upon its suppression, we performed in vivo xenograft assay. Intravenously and subcutaneously injected nude mice with SKOV-3 cells (control groups), were treated with control and CARF siRNAs, as shown in the treatment regime (Fig. 7a). On day 40, observation of their liver and lungs revealed metastatic foci/sacs in control and CARF-enriched cell-injected group, however, their size was found to be larger in the latter (Fig. 7a). Of note, the group given intermittent injections of CARF siRNA had normal liver and lungs (Fig. 7a). Concomitantly, tumors given intratumoral CARF siRNA injections harbored smaller-size tumors compared to the control, while mice injected with CARF-enriched cells raised larger tumors (Fig. 7b). Mice body-weight and tumor-weight analyses revealed that CARF enrichment promoted aggressive tumors and its knockdown led to suppression of their growth (Fig. 7a, b).

We next employed CARF targeting oncolytic adenovirus with lung metastatic xenograft model using luciferase-expressing invasive lung cell line, i.e., A549. As shown in Fig. 7c, treatment of RdB-shCARF resulted in reduced metastatic progression in lungs. At 21 days post injection, the luciferase signal from the metastatic lung tumors of mice treated with RdB-shCARF (2.4 × 10^7^ p/s) was significantly attenuated in comparison to other groups demonstrating 186.2-, or 82.2-fold greater therapeutic efficacy than PBS (4.5 × 109 p/s), or RdB (2.0 × 109 p/s), respectively (Fig. 7c, d), these data demonstrated and endorsed the physiological relevance of CARF in metastases and its inhibition by CARF knockdown.

## DISCUSSION

CARF has been established as an essential protein that poses two-way control on cell proliferation by regulation of ARF-p53-HDM2-p21^WAF1^ signaling^15–18^. Overexpression of CARF in ARF-dependent or independent manner was shown activate and stabilize p53 function causing pre-mature senescence^16,17^. On the other hand, excessively enriched/super-high level of CARF expression promoted proliferation and malignant properties of cancer cells^18,19^. In the present study, enrichment of CARF (gene and transcript) levels in cancer patient datasets/tumor samples suggested clinical relevance of CARF upregulation with cancer metastasis (Figs. 1 and 2). Furthermore, patients with amplified CARF marked higher amplification level of several markers involved in metastasis and angiogenesis (Fig. 1d). In vitro analyses of markers involved in EMT, invasion, and migration^21,22^ (Fig. 3e–h; Figure S2) exhibited their upregulation in CARF-enriched both U2OS and SKOV-3 cells. The clinical readouts and molecular analyses conferred that enrichment of CARF is tightly associated with EMT, metastasis and invasion. CARF amplification and enriched expression levels showed an association with β-catenin across tumor datasets (Figures S3). Of note, CARF-enriched cells showed a distinct nuclear enrichment and transcriptional activation of β-catenin as supported by increased expression of its gene targets including SNAIL1, TWIST1, SNAI2, ZEB1, and effectors proteins. By molecular analyses and use of specific inhibitors, we determined that CARF activates Wnt/β-catenin signaling through kinase pathway.

The above data demonstrated a tight correlation and suggested an important role of CARF in EMT progression. siRNA-mediated CARF knockdown in SKOV-3, HT-1080, MDA-MB-231 invasive cells caused inhibition of cell migration and invasive characteristics. Furthermore, reduced mesenchymal (vimentin, N-cadherin, fibronectin) and elevated epithelial (E-cadherin) properties in these cells supported that CARF could be a potential therapeutic target in reversing the EMT process. Of note, whereas CARF enrichment caused nuclear translocation of β-catenin, its abolition was marked in CARF-compromised cells demonstrating that CARF has essential role in activation of β-catenin signaling involved in EMT. The data was endorsed by downregulation of several β-catenin-regulated proteins including, survivin, c-Myc, Cyclin D1, Wnt1, and SMAD2/3. Furthermore, β-catenin interacting proteins required for active transcriptional complex (AXIN2, DVL, TCF4 & LEF1) and effectors (Cyclin D1, c-Myc, survivin) showed increase in CARF-enriched and decrease in CARF-compromised cells.

Recently, Fan et al.^29^, using an oncogenic Ras model of hepatocellular carcinoma (HCC), reported that CARF promotes β-catenin/TCF activation by inhibition of β-catenin and ICAT interaction. He et al.^30^ reported a small-molecule inhibitor of CARF. Using in vitro and embryonic developmental model of zebra fish, they showed that suppression of CARF led to impaired embryonic development, hematopoietic and caudal fin regeneration. The study showed a direct CARF–Dvl interaction that facilitated TCF/β-catenin transcriptional activity^30^. In continuation with our earlier studies that demonstrated that CARF is an essential and dual regulator of cell proliferation^14–19^. We provide clinical relevance of CARF upregulation in malignant progression of cancer and mechanism of its role in EMT, migration and invasion (Fig. 7e). Of note, frequencies of CARF amplification with that of β-catenin, Dvl2, TCF4, and c-Jun across patient datasets implying a possible association of CARF in stabilizing Dvl-c-Jun-β-catenin-TCF transcriptional complex as was also hypothesized by He et al.^30^ Markedly, CARF-induced transcriptional activation of TCF/β-catenin could be reversed by siRNA-mediated CARF silencing in in vitro as well as in vivo models suggesting that targeting of CARF has therapeutic potential for treatment of metastatic and invasive cancers.

Taken together, we report that CARF (i) amplification is clinically relevant to cancer invasiveness and metastasis, (ii) facilitates EMT and metastatic progression via activation of Wnt/β-catenin signaling and (iii) is a strong therapeutic target for malignant and aggressive cancers.

## Materials and methods

### Reagents

Antibodies purchased from various sources as follows: β-actin-HRP, c-Myc and uPA, (Abcam); N-cadherin, SMAD2/3, p21^WAF1^, and p53 (Cell Signaling); β-catenin, Wnt1, E-cadherin, Survivin, Vimentin, Fibronectin, Kinesin, VEGF, MMP2, MMP9, Cyclin D1, hnRNP-K, TGF- β1, and rabbit-/mouse-HRP (Santa Cruz); and mouse/rabbit Alexa Fluor 488- and/or 568-conjugated secondary antibodies and Hoechst 33342 (Invitrogen) additional details provided in Supplementary Information (Table S1). Anti-CARF antibody (Clone FL-A10) was raised indigenously in the laboratory^15^. CARF siRNA (Silencer® Select & In vivo Ready Oligos) were purchased from Thermo Fisher. Wnt and β-catenin inhibitors, i.e. IWP2 (Santa Cruz), Pyrvinium pamoate (PP) and iCRT14 (Sigma-Aldrich) were purchased. Tissue microarray slides with embedded clinical tumor (various normal/cancer and breast cancer samples of histology, stage, age) were procured from SuperBioChip (Tissue-Array, South Korea) Laboratories.

### TCGA, Oncomine, and HPA analyses

Genomic amplification, transcript and protein expression analysis of CARF in clinical datasets was performed using TCGA (http://www.cbioportal.org/index.do), Oncomine (https://www.oncomine.org/), and Human Protein Atlas (HPA; http://www.proteinatlas.org/) respectively; while pathway genes-lists were obtained from KEGG (http://www.genome.jp). Detailed procedures of above analyses are provided in supplementary methods.

### Cell culture

Human cancer (ovarian and cervical adenocarcinoma—SKOV-3, HeLa (RIKEN cell bank)); osteosarcoma U2OS, SaOS-2 (JCRB Cell Bank); breast carcinoma—MCF-7, MDA-MB-231 and T47D (DS Pharma Biomedical); melanoma—G361 and fibrosarcoma-HT-1080 (HSRRB, Japan); hepatocarcinoma—HUH-6, HUH-7, colon carcinoma—DLD-1 and lung carcinoma—H-1299, A549 (JCRB Cell Bank) cell lines were grown in DMEM, while human neuroblastoma (IMR-32; HSRRB, Japan) cells cultured in MEM medium. Cultures were maintained as described previously^18,28^. Cell lines were authenticated by either STR-PCR or Isozyme profiling at their respective sources. Cell stocks were cryopreserved (in multiple vials) in liquid Nitrogen (LN_2_) and were regularly revived from the original stocks to avoid genetically instable cells that may arise due to prolong culture. Cells were treated with inhibitors, IWP2 (0.5 μM for U2OS and 5 μM for SKOV-3), PP (2.5 nM for U2OS and 50 nM for SKOV-3), and iCRT14 (1.5 μM for U2OS and 10 nM SKOV-3) for 24 h.

### CARF-enriched and -compromised cells

Control (pCX^Neo^, empty vector) and CARF (full length CARF-tagged with GFP)-enriched (infected with retroviral titter (1:1 ratio), viz super-higher expression of CARF) cells were generated and maintained in medium supplemented with G418 (100 μg/mL) as described earlier^18^. For CARF knockdown, CARF Silencer® Select siRNA was transfected with Lipofectamine® RNAiMAX (Thermo) using standard procedure as previously described^19,28^. Optimum siRNA concentration (10–200 pM, 72 h) was determined by viability (MTT, Roche) assay and the nontoxic doses (20–50 pM, 48 h) were used for transient transfections in U2OS, SKOV-3, MBA-MB-231, and HT-1080 cells.

### Reverse transcription (RT)-PCR, Real-Time PCR, and Genomic PCRs

Semiquantitative RT and Real-Time PCRs were performed as described earlier^18,31^. Genomic PCR was performed on a panel of normal and metastatic breast cancer cell lines (American Type Culture Collection, ATCC) as described earlier^32^ using CARF-specific primer sets (Table S2).

### Immunohistochemistry (IHC)

CARF expression in tissue microarray was examined as described earlier^19^. Quantitation of expression level (stained area in images) was performed using Image J software (National Institute of Health, Bethesda, MA) and represented as percentage of stained area.

### Immunoblotting

Protein extraction and immunoblotting with specific antibodies (as indicated) was performed as described previously^18^. Quantitation of signals (performed with the Image J) of three independent blots (normalized with β-actin, an internal loading control) was represented as percentage expression.

### Immunofluorescence (IF)

Immunofluorescence staining was performed as described earlier^18^ using Zeiss Axioplan 2 microscope (with a Zeiss AxioCam HRc camera) and confocal laser scanning microscope (LSM510, Carl Zeiss). To resolve the subcellular localization of protein, the images were analyzed with the Imaris (Bitplane) software.

### Invasion and wound-healing assay

Invasion assay was performed by seeding 5 × 10^4^ cells in upper chamber of Matrigel (BD BioSciences) coated surface in a 12-well plate. The procedure, staining and image acquisition was performed as earlier described^19^. Wound-healing assay with different (CARF-enriched/-silenced U2OS, SKOV-3, MBA-MB-231 and HT-1080) cells for 48 and 72 h was performed as described earlier^18^. Phase contrast images (×10 magnification) at different time-points were captured and quantitated for scratched area.

### In vivo xenograft assays

Five- to six-weeks-old female nude mice were injected subcutaneously and intravenously with 2.5 × 10^6^ control (2 groups—3 mice each; post-2 weeks for subsequent treatments, mice were randomized into 2 groups for subsequent Control and CARF siRNA treatments) and CARF-enriched (1 group; 3 mice) SKOV-3 cells. CARF-targeting adeno-oncolytic virus (RdB-shCARF) was generated^19^ and employed with lung metastasis model (A549 cells, expressing firefly luciferase) as described earlier^33^. Detailed regimes of treatment, doses and tumor growth monitoring are provided in supplementary methods. All observations were performed, updated and duly discussed to avoid blinding on allocation/accessing the experimental outcome. This study was carried out in strict accordance with the recommendations in the Animal Experiment Committee, Safety and Environment Management Division, National Institute of Advanced Industrial Science & Technology (AIST), Japan (Experimental plan approval #2012-025).

### Statistical analysis

All quantitative experiments were performed in triplicate. Data values are represented as mean ± SEM of three individual replicates. The two-tailed Student’s *t*-test or Mann–Whitney *U*-test (non-parametric), whichever applied, was enrolled to calculate the degree of significance among the control and test group. The statistical significance was expressed as *p*-value ≤ 0.05; and represented by *<0.05, **<0.01, ***<0.001, while NS marked the insignificant correlation^34^.

## Electronic supplementary material

Suppl. Materials
