# Supplementary material for: CARF enrichment promotes epithelial–mesenchymal transition via Wnt/β-catenin signaling: its clinical relevance and potential as a therapeutic target
Source: Oncogenesis. 2018 May 11;7(5):39. doi: 10.1038/s41389-018-0048-4 (PMC5945778; doi:10.1038/s41389-018-0048-4)
Supplement: Supplementary file 1 — Suppl. Materials [file 41389_2018_48_MOESM1_ESM.pdf]

SUPPLEMENTARY INFORMATION -

CARF Enrichment Promotes Epithelial-mesenchymal Transition via Wnt/β-catenin Signaling: Its Clinical Relevance and Potential as a Therapeutic Target

Rajkumar S. Kalra[1], Anupama Chaudhary[1], A-Rum Yoon[2], Priyanshu Bhargava[1], Amr Omar[1], Sukant Garg[1], Chae-Ok Yun[2], Sunil C. Kaul[1], and Renu Wadhwa[1]

[1]Drug Discovery and Assets Innovation Lab, DBT-AIST International Laboratory for Advanced Biomedicine (DAILAB), DAICENTER, National Institute of Advanced Industrial Science & Technology (AIST), Tsukuba, Ibaraki 305-8565, Japan.

[2]Department of Bioengineering, College of Engineering, Hanyang University, Seongdong-gu, Seoul, Korea.

Summary - Supplementary Information includes details of employed supplementary methods, legends to supplementary figures, details of antibodies used, and primer sequences used for Q-PCR, RT (Reverse Transcriptase)-PCR and genomic-PCR amplifications.

Supplementary Information: List of contents -

Supplementary methods

Legends to the Supplementary Figures -

Supplementary Figure 1

Supplementary Figure 2

Supplementary Figure 3

Supplementary Figure 4

Supplementary Figure 5

Supplementary Figure 6

Supplementary Figure 7

Supplementary Figure 8

Supplementary Figure 9

Table S1: Detail of antibodies used in immunoblotting and immunofluorescence.

Table S2: Detail of primers used for RT-PCR amplifications.

SUPPLEMENTARY METHODS-

TCGA, Oncomine and HPA analyses

Patient data information for CARF genomic amplifications, and its expression levels (mRNA expression, RNA-Seq & V2 values) were analyzed across datasets (data sets of circulating/blood-born, and neurological malignancies omitted amongst metastatic analyses) using TCGA portal (http://www.cbioportal.org/index.do). Analyses of amplification frequencies of genes listed in TCGA-defined Invasion/Metastases and Angiogenesis datasets were taken to examine their status in altered (*n=10*) and unaltered (*n=97*) patient cohort for CARF amplification in Prostate cancer (Neuroendocrine, NEPC; Trento/Cornell/Broad 2016) dataset, retaining highest % of CARF amplification.

Also, NEPC dataset was explored to retrieve expression data (*p value*, Log odds ratio) of genes involved in Wnt/β-catenin signaling, whereas gene list was obtained from KEGG (http://www.genome.jp/dbget-bin/get_linkdb?-t+8+path:map04310) pathways. Gene expression data of customized β-catenin gene (direct and indirect) targets was also analyzed in NEPC dataset. Furthermore, taking CTTNB1, TGFB1, EGFR, ERBB2 and NOTCH1 amplification frequencies in in top 10 datasets; status of CARF/CDKN2AIP respectively was analyzed in these datasets. Similarly, information on amplification frequencies of the β-catenin interactors CTTNB1, DVL2, TCF4, LEF1 and JUN of transcriptional complex, and other key genes involved in Wnt/β-catenin signaling also retrieved in top 10 datasets, and was analyzed for CARF status. The Oncomine (https://www.oncomine.org/), an online portal of cancer microarray database was used to analyze CARF expression across normal and tumor types using differential expression algorithm. Expression data was plotted as log2 median-centered intensity. The Human Protein Atlas (HPA; http://www.proteinatlas.org/), an antibody-based proteomic expression database was explored to examine CARF protein expression in normal versus cancer tissues sections. The immunohistochemical data from the HPA was retrieved and quantitated for CARF expression in various normal, cancer and breast cancer sample sections (normal, lobular and ductal carcinomas).

In vivo xenograft assays

Mice injected subcutaneously were observed and given intra-tumor (50μL, 50μM) Control and Ambion® In Vivo CARF siRNA (Thermo Fisher Scientific) injections post 2 weeks in 2 control groups respectively. Whereas, mice injected intravenously given control and CARF siRNA (100μL, 50μM) injections intravenously in the tail vein. Control and CARF siRNA injections were repeated twice a week for the next 4.5 weeks. Tumor sizes of in subcutaneously injected mice were measured twice a week on above time points. Mice injected intravenously and subcutaneously were sacrificed by cervical dislocation on 40th day and examined for metastatic foci/sacs in liver and lungs, while subcutaneous tumors were collected in the latter group. To assess the anti-metastatic effect on CARF knockdown, a CARF oncolytic (RdB-shCARF) metastatic lung cancer model was established using A549 cells. A total of 1×10^6 firefly luciferase-expressing

A549 cells were injected via tail vein to 6-week old male nude mice (Charles River Korea Inc.). After 4 weeks, bioluminescence imaging was measured to confirm the acquisition of orthotopic lung tumors. Mice were subsequently anesthetized in a chamber containing 2% isoflurane and given intra-peritoneal D-luciferin (150 mg/kg; Caliper, Hopkinton, MA) injections. Metastatic lung tumor-harboring mice were randomized into 3 groups (≥4 mice each group) for subsequent treatments, and injected three times every other day with 200 µL of PBS, RdB (2 × 1010 VP), or RdB-shCARF (2 × 1010 VP). The luminescent images were obtained from the anesthetized mice using the IVIS imaging system (Xenogen, Alameda, CA). In vivo bioluminescence signal intensity was obtained as photons acquired per second (photons/second [p/s]) from a body region of interest.[24] Tumor growth was measured every week by bioluminescence imaging, following the first treatment.

LEGENDS TO THE SUPPLEMENTARY FIGURES-

Supplementary Figure 1. a, Gene expression data showing enrichment of CARF mRNA levels in different TCGA cancer datasets; RNA-Seq expressions shown in log10 scale. **b**, Log2 CARF expressions as analyzed in normal breast duct, invasive lobular and invasive ductal carcinomas in Thurashvili Breast dataset using Oncomine. **c**, Percentage CARF mRNA enrichment in cancer cell lines having CARF amplification in Cancer Cell Line Encyclopedia (CCLE; Novartis/Broad 2012) dataset of TCGA includes number of circulate/blood-born (#1) and invasive cell lines derived either from primary (#2) or secondary (#3) metastatic tumor site.

Supplementary Figure 2. a, Immunoblots showing increased expressions of mesenchymal (n-cadherin, fibronectin, kinesin, and dynein) and invasive (MMP2, MMP9, hnRNP-K, and VEGF) markers in SKOV-3 CARF-GFP cells. **b**, Immunostaining showing increase in vimentin and reduced E-cadherin expression in SKOV-3 CARF-GFP cells, as shown upper; while other mesenchymal (n-cadherin and fibronectin) markers also increased in these cells, shown below. **c**, Immunostaining showing increase in hnRNP-K expression in U2OS CARF-GFP cells.

Supplementary Figure 3. Percentage of matched genomic CARF amplifications as analyzed in individual top 10 patient datasets having CTNNB1 **a**, TGFB1 **b**, EGFR **c**, ERBB2 **d**, and NOTCH1 **e** amplifications, represented by opposite red bars from axis base. **f**, Gene expression of the candidate genes involved in Wnt/β-catenin signaling as analyzed in NEPC (Trento/Cornell/Broad 2016) dataset; p value and log odds ratio showing expressions.

Supplementary Figure 4. Log odds ratio showing gene expression values of β-catenin targets involved in EMT as analyzed in NEPC (Trento/Cornell/Broad 2016) dataset. Graph showing gene expression values of its indirect (categorized into repressors and activators) target genes in the dataset, as listed on the right are shown next.

Supplementary Figure 5. a, Percentage of genomic amplifications of CARF matched with individual top 10 patient datasets having amplifications in CTNNB1, TCF4, LEF1, and JUN genes; while, **b**, showing matched amplification frequencies of CARF with number of β-catenin gene targets including TDG, SIAH1, CSNK1A1, BTRC, FRAT1, MAP3K7, SMAD4, and TLE1 in analyzed NEPC (Trento/Cornell/Broad 2016) dataset.

Supplementary Figure (6). a, Transcript levels of the transcription factors (SNAIL1, SNAIL2, ZEB1, and TWIST1) and CDH1 gene validated in SKOV-3 control and CARF-GFP cells; while **b**, showing transcript levels of β-catenin target gene (WNT1, CTNBB1, DVL2, AXIN2, TCF4, and LEF1) in these cells; while **c-d**, showing transcript levels of TGFβ1, its receptor TGFBR1, AKT, and FAK in U2OS and SKOV-3 vector control and CARF-GFP cells respectively; quantitation shown in fold change on the right. **e**, Immunoblots showing increase in β-catenin and its target proteins *viz.* survivin, cyclin D1, and SMAD-2/3 in SKOV-3 CARF-GFP cells.

Supplementary Figure 7. a, Immunoblots showing expression levels of β-catenin and its targets proteins in Control (NT), IWP2 and PP treated SKOV-3 pCXNeo Control and CARF-GFP cells. **b**, Immunoblots for β-catenin, survivin and SMAD2/3 in iCRT14

treated cells; quantitation is shown on the right. **c**. β-catenin immunostaining in IWP2 and PP treated SKOV-3 pCXNeo Control and CARF-GFP cells.

Supplementary Figure 8. a, Immunoblots showing CARF knockdown in invasive fibrosarcoma (HT-1080) and breast carcinoma (MDA-MB-231) cell lines with CARF siRNA doses (10-50 pMoles). **b**, wound healing assay showing a progressively reduced cell migration on increasing CARF siRNA doses (10-50 pMoles), as observed at 48 h time point in MDA-MB-231 cells; while, **c-d**, showing cell morphology and quantitation of percentage scratched or covered area at 48 h time point in these cells upon CARF knockdown.

Supplementary Figure 9. a, mRNA expression levels of the transcription factors (SNAIL1, SNAIL2, ZEB1, and TWIST1) and CDH1, ACTA2 gene validated in control and CARF siRNA transfected U2OS cells; while **b**, showing transcript levels of β-catenin target gene (WNT1, CTNBB1, DVL2, AXIN2, TCF4, LEF1, and CCND1) in these cells. **c**, Transcript levels of TGFβ1, TGFBR1, AKT, and FAK in control and CARF siRNA transfected U2OS cells; quantitation is plotted as fold change on the right. **d**, Immunostaining showing reduced nuclear SMAD-2/3 expression in CARF knockdown cells, as compared to transfected control U2OS cells.

Supplementary Table S1: Detail of antibodies used in immunoblotting and immunofluorescence assays.

Antibody	Source	Catalog number
E-cadherin	Santa Cruz	sc-21791
N-cadherin	Cell Signaling	#13116
Fibronectin	Santa Cruz	sc-52331
Kinesin (H1)	Chemicon	#MAB1613
Vimentin (H-84)	Santa Cruz	sc-5565
MMP2	Santa Cruz	sc-10736
MMP-9 (G657)	Cell Signaling	#2270S
hnRNP-K	Santa Cruz	CS#4675S
uPA	Abcam	ab169754
β-catenin	Santa Cruz	sc-7963
Survivin	Santa Cruz	sc-17779
Cyclin D1	Santa Cruz	sc-450
TGF-β1	Santa Cruz	sc-146
SMAD2/3	Cell Signaling	#8685
Wnt1	Santa Cruz	sc-514531
c-Myc (Y69)	Abcam	ab32072
VEGF(A-20)	Santa Cruz	sc-152
Dynein	Abcam	ab23905
β-actin-HRP	Abcam	ab49900
Ant-Mouse-HRP	Santa Cruz	sc-2005
Ant-Rabbit-HRP	Santa Cruz	sc-2004
Anti-Mouse Alexa Fluor® 488	Thermo-Fisher	A11029
Anti-Mouse Alexa Fluor® 594	Thermo-Fisher	A11032
Anti-Rabbit Alexa Fluor® 488	Thermo-Fisher	A11034
Anti-Rabbit Alexa Fluor® 594	Thermo-Fisher	A11037

Antibody produced indigenously (usage details below)-		
Rabbit anti-CARF	Immunoblotting	Immunofluorescence
	1:2500	1:100

Supplementary Table S2: Details of primers used for Genomic, Quantitative and RT-PCR amplifications.

Details of primers used for Genomic-PCR amplifications

Primers	*Primer/Nucleotides Sequence from 5′-3′*	*Annealing temp*
CARF_F	5′-GTTCTTGGCGGTTGGTTCAC-3′	60°C
CARF_R	5′-TCCCTCTTTTCTTCACCTTGGC-3′	
GAPDH_F	5′-CATCCCTTCTCCCCACACAC-3′	58°C
GAPDH_R	5′-AGTCCCAGGGCTTTGATTTG-3′	

Details of primers used for qPCR amplifications

Primers	*Primer/Nucleotides Sequence from 5′-3′*	*Annealing temp*
CARF_F	5′-TCAAAGTGACAGATGCTCCAAC-3′	60°C
CARF_R	5′-GGTTTTTGCGTGATCTTGCTC-3′	
CK8-F	5′-CAG AAGTCCTACAAGGTGTCCA-3′	60°C
CK8-R	5′-CTCTGGTTGACCGTAACTGCG-3′	
CK14-F	5′-AGGACCTGAAGAGCAAGATC-3′	60°C
CK14-R	5′-TCCTTGAGGCTCTCAATCTG-3′	
CK18-F	5′-ATGACACCAACATCACAAGG-3′	60°C
CK18-R	5′-ATCCACTTCCACAGTCAATC-3′	
WNT3A-F	5′-CAAGATTGGCATCCAGGAGT-3′	60°C
WNT3A-R	5′-TCCCTGGTAGCTTTGTCCAG-3′	
GAPDH-F	5′-TGGAAATCCCATCACCATCT-3′	60°C
GAPDH-R	5′-TTCACACCCATGACGAACAT-3′	

Details of primers used for RT (Reverse Transcriptase) - PCR amplifications

Primers	*Primer/Nucleotides Sequence from 5'-3'*	*Annealing temp*
SNAI1_F	5′-CAGACCCACTCAGATGTCAA-3′	60°C
SNAI1_R	5′-CATAGTTAGTCACACCTCGT-3′	
SNAI2_F	5′-GGTCAAGAAGCATTTCAAC-3′	60°C
SNAI2_R	5′-GGTAATGTGTGGGTCCGA-3′	
ZEB1_F	5′-TTCAAACCCATAGTGGTTGCT-3′	60°C
ZEB1_R	5′-TGGGAGATACCAAACCAACTG-3′	
TWIST_F	5′-GGGAGTCCGCAGTCTTAC-3′	60°C
TWIST_R	5′-CCTGTCTCGCTTTCTCTTT-3′	
WNT1_F	5′-TGGCCGATGGTGGGGTATTGTGA-3′	66°C
WNT1_R	5′-CGGCCTGCCTCGTTGTTGTGAA-3′	
CTNNB1_F	5′-TGATGGAGTTGGACATGGCCATGG-3′	60°C
CTNNB1_R	5′-CAGACACCATCTGAGGAGAACGCA-3′	
DVL2_F	5′-CCGTCATGTGCTTGCTCTTA-3′	60°C
DVL2_R	5′-TGGAGGAGGAGGTCACATTC-3′	
AXIN2_F	5′-GTTGGTGACTTGCCTCCCGGACCC-3′	60°C
AXIN2_R	5′-GAGTGTAAGGACTTGGTCCACCGG-3′	
TCF4_F	5′-CCGATGACGAGGGTGATGAG-3′	60°C
TCF4_R	5′-CCGAGGACACCTTCTCTTCC-3′	
LEF1_F	5′-TTCAAGGACGAGGGCGAT-3′,	60°C
LEF1_R	5′-TGTACCCGGAATAACTCG-3′	
CDH1_F	5′-GAGAACGCATTGCCACATAC-3′	60°C
CDH1_R	5′-GAAGAGCACCTTCCATGACA-3′	
TGFB1_F	5′-TGGCGATACCTCAGCAACC-3′	61°C
TGFB1_R	5′-CTCGTGGATCCACTTCCAG-3′	
TGFBR1_F	5′-ACGGCGTTACAGTGTTCTG-3′	63°C
TGFBR1_R	5′-GGTGTGGCAGATATAGACC-3′	
AKT_F	5′-GCAGCACGTGTACGAGAAGA-3′	60°C
AKT_R	5′-GGTGTCAGTCTCCGACGTG-3′	
FAK_F	5′-GGTGCAATGGAGCGAGTATT-3′	60°C
FAK_R	5′-GCCAGTGAACCTCCTCTGA-3′	
ACTA2_F	5′-ACCCACAATGTCCCCATCTA-3′	60°C
ACTA2_R	5′-TGATCCACATCTGCTGGAAG-3′	
CCND1_F	5′-ATGGAACACCAGCTCCTGTGCTGCG-3′	60°C
CCND1_R	5′-TCCAGGTAGTTCATGGCCAGCGGG-3′	
CARF_F	5′-TCAAAGTGACAGATGCTCCA-3′	60°C
CARF_R	5′-CGTTGAACTGTTTTCCTGCT-3′	

Sup Fig. 1

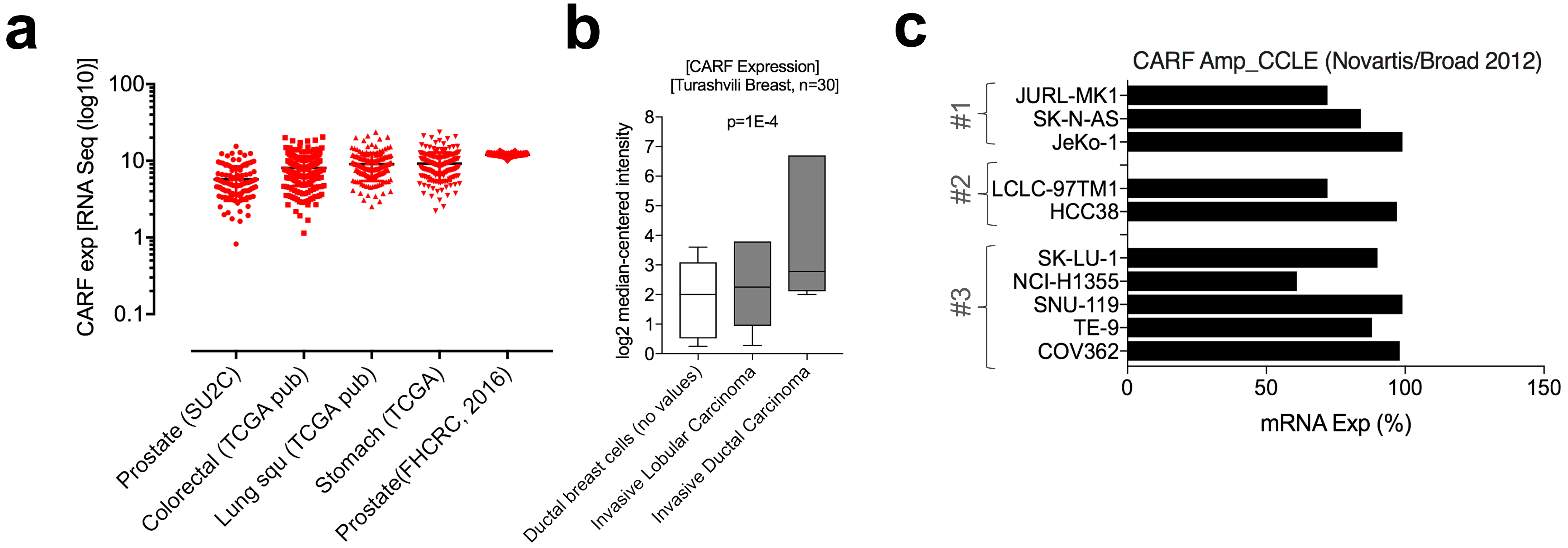

a
CARF exp [RNA Seq (log10)]
100
10
1
0.1
Prostate (SU2C)
Colorectal (TCGA pub)
Lung squ (TCGA pub)
Stomach (TCGA)
Prostate(FHCRC, 2016)
b
[CARF Expression]
[Turashvili Breast, n=30]
p=1E-4
log2 median-centered intensity
8
7
6
5
4
3
2
1
0
Ductal breast cells (no values)
Invasive Lobular Carcinoma
Invasive Ductal Carcinoma
c
CARF Amp_CCLE (Novartis/Broad 2012)
#1
JURL-MK1
SK-N-AS
JeKo-1
#2
LCLC-97TM1
HCC38
#3
SK-LU-1
NCI-H1355
SNU-119
TE-9
COV362
0
50
100
150
mRNA Exp (%)

Sup Fig. 2

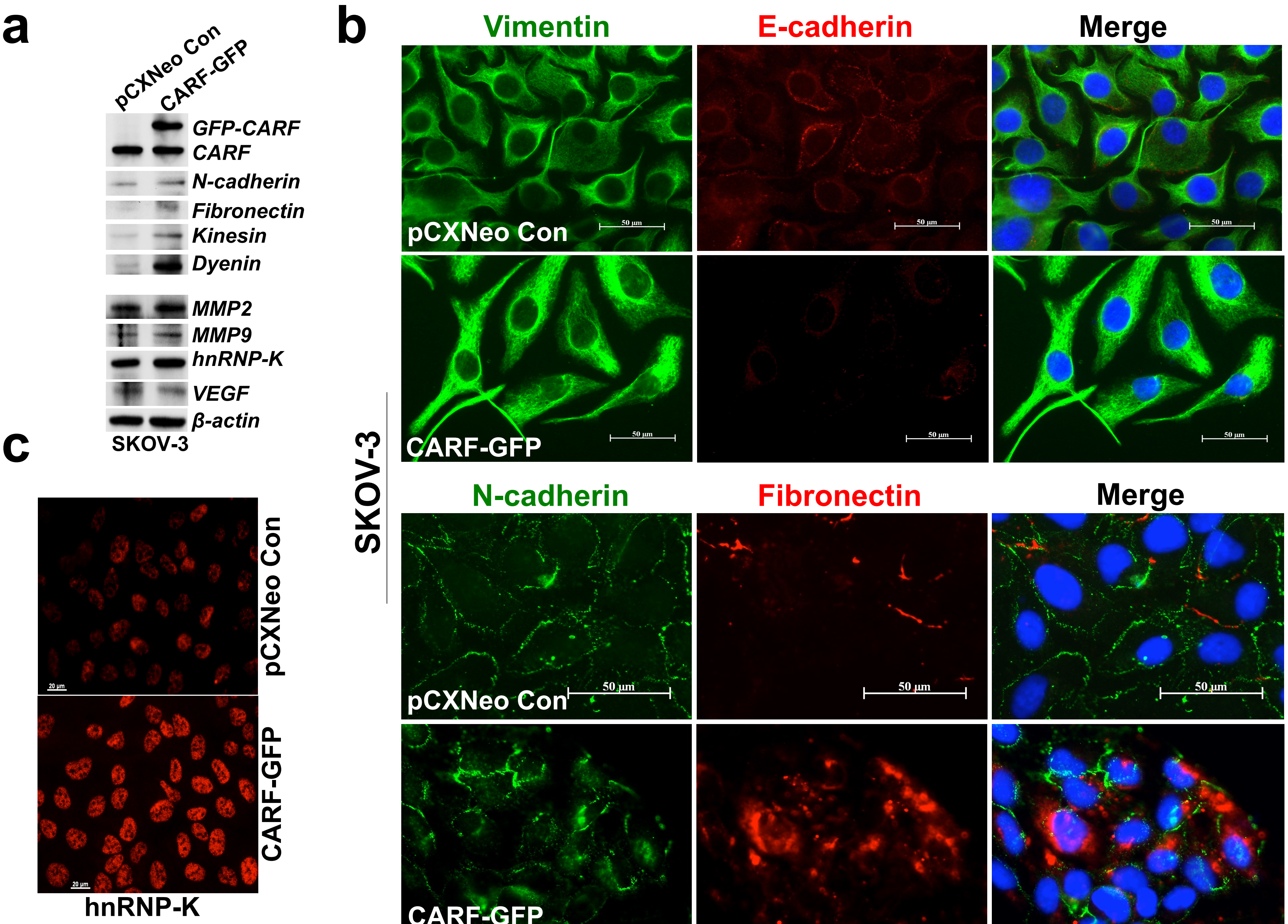
a
pCXNeo Con
CARF-GFP
GFP-CARF
CARF
N-cadherin
Fibronectin
Kinesin
Dyenin
MMP2
MMP9
hnRNP-K
VEGF
β-actin
SKOV-3
b
Vimentin
E-cadherin
Merge
pCXNeo Con
CARF-GFP
50 µm
SKOV-3
N-cadherin
Fibronectin
Merge
pCXNeo Con
CARF-GFP
c
pCXNeo Con
CARF-GFP
20 µm
hnRNP-K

Sup Fig. 3

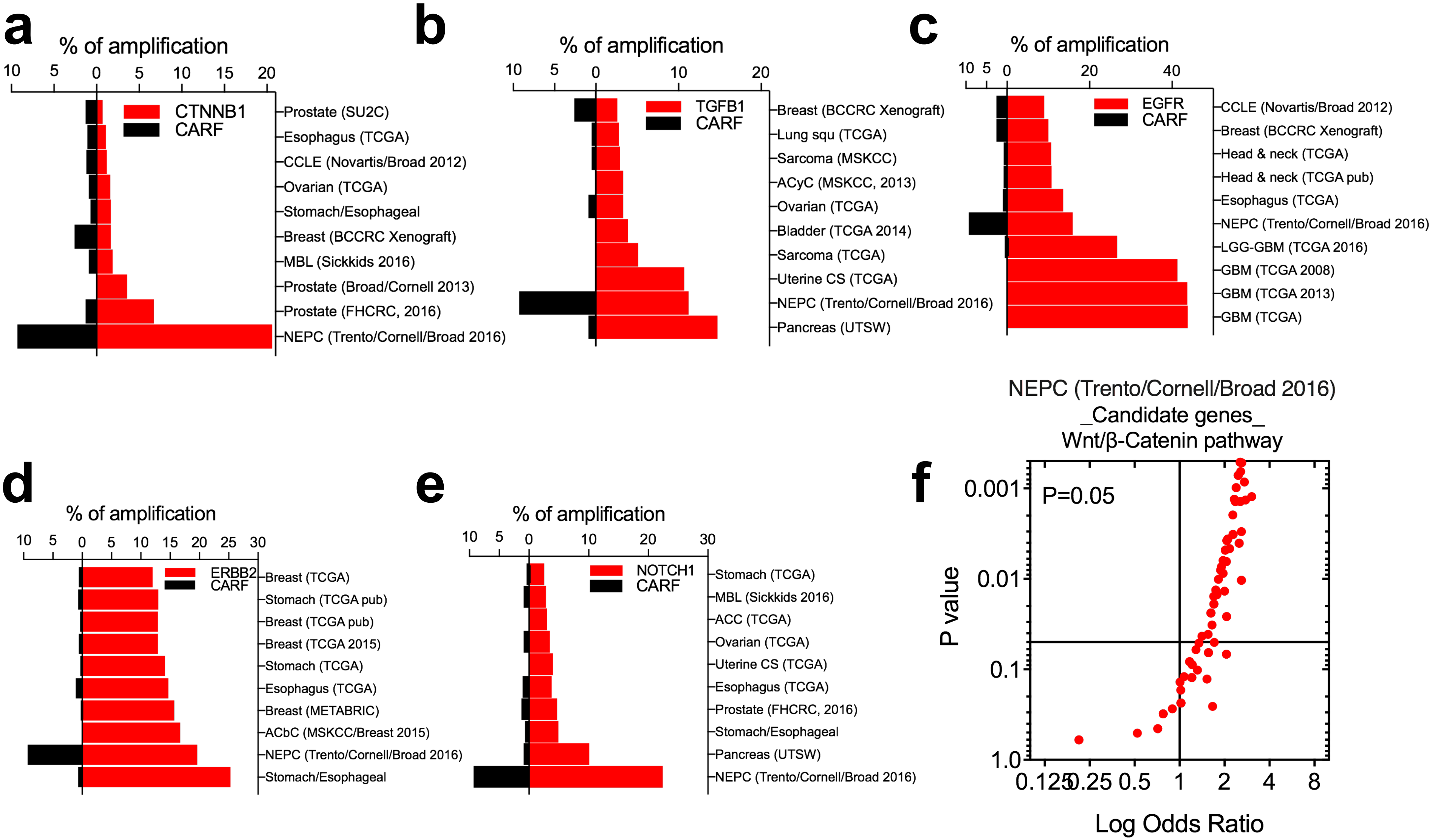
a
% of amplification
10 5 0 5 10 15 20
CTNNB1
CARF
Prostate (SU2C)
Esophagus (TCGA)
CCLE (Novartis/Broad 2012)
Ovarian (TCGA)
Stomach/Esophageal
Breast (BCCRC Xenograft)
MBL (Sickkids 2016)
Prostate (Broad/Cornell 2013)
Prostate (FHCRC, 2016)
NEPC (Trento/Cornell/Broad 2016)
b
% of amplification
10 5 0 10 20
TGFB1
CARF
Breast (BCCRC Xenograft)
Lung squ (TCGA)
Sarcoma (MSKCC)
ACyC (MSKCC, 2013)
Ovarian (TCGA)
Bladder (TCGA 2014)
Sarcoma (TCGA)
Uterine CS (TCGA)
NEPC (Trento/Cornell/Broad 2016)
Pancreas (UTSW)
c
% of amplification
10 5 0 20 40
EGFR
CARF
CCLE (Novartis/Broad 2012)
Breast (BCCRC Xenograft)
Head & neck (TCGA)
Head & neck (TCGA pub)
Esophagus (TCGA)
NEPC (Trento/Cornell/Broad 2016)
LGG-GBM (TCGA 2016)
GBM (TCGA 2008)
GBM (TCGA 2013)
GBM (TCGA)
d
% of amplification
10 5 0 5 10 15 20 25 30
ERBB2
CARF
Breast (TCGA)
Stomach (TCGA pub)
Breast (TCGA pub)
Breast (TCGA 2015)
Stomach (TCGA)
Esophagus (TCGA)
Breast (METABRIC)
ACbC (MSKCC/Breast 2015)
NEPC (Trento/Cornell/Broad 2016)
Stomach/Esophageal
e
% of amplification
10 5 0 10 20 30
NOTCH1
CARF
Stomach (TCGA)
MBL (Sickkids 2016)
ACC (TCGA)
Ovarian (TCGA)
Uterine CS (TCGA)
Esophagus (TCGA)
Prostate (FHCRC, 2016)
Stomach/Esophageal
Pancreas (UTSW)
NEPC (Trento/Cornell/Broad 2016)
f
NEPC (Trento/Cornell/Broad 2016)
Candidate genes
Wnt/β-Catenin pathway
P=0.05
P value
0.001
0.01
0.1
1.0
0.125 0.25 0.5 1 2 4 8
Log Odds Ratio

Sup Fig. 4

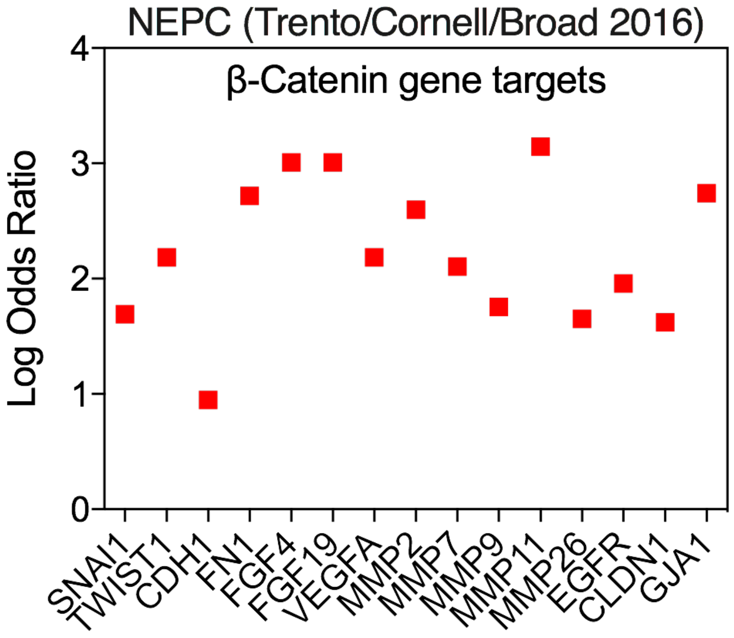

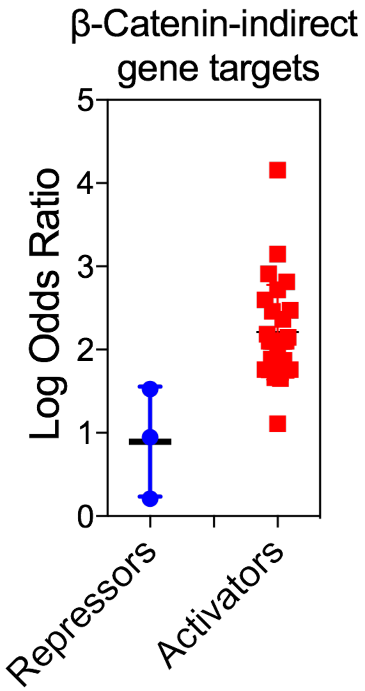

β-catenin direct targets (EMT)
SNAI1
TWIST1
CDH1
FN1
FGF4
FGF19
FGF20
VEGFA
MMP2
MMP7
MMP9
MMP11
MMP26
EGFR
CLDN1
GJA1

β-catenin indirect targets (EMT)	
Repressors	*Activators*
CDH1	VTN
OCLN	CDH2
TJP1	COL1A1
	TWIST2
	ZEB1
	ZEB2
	NEDD9
	FOXC2
	FOXO3A

Sup Fig. 5

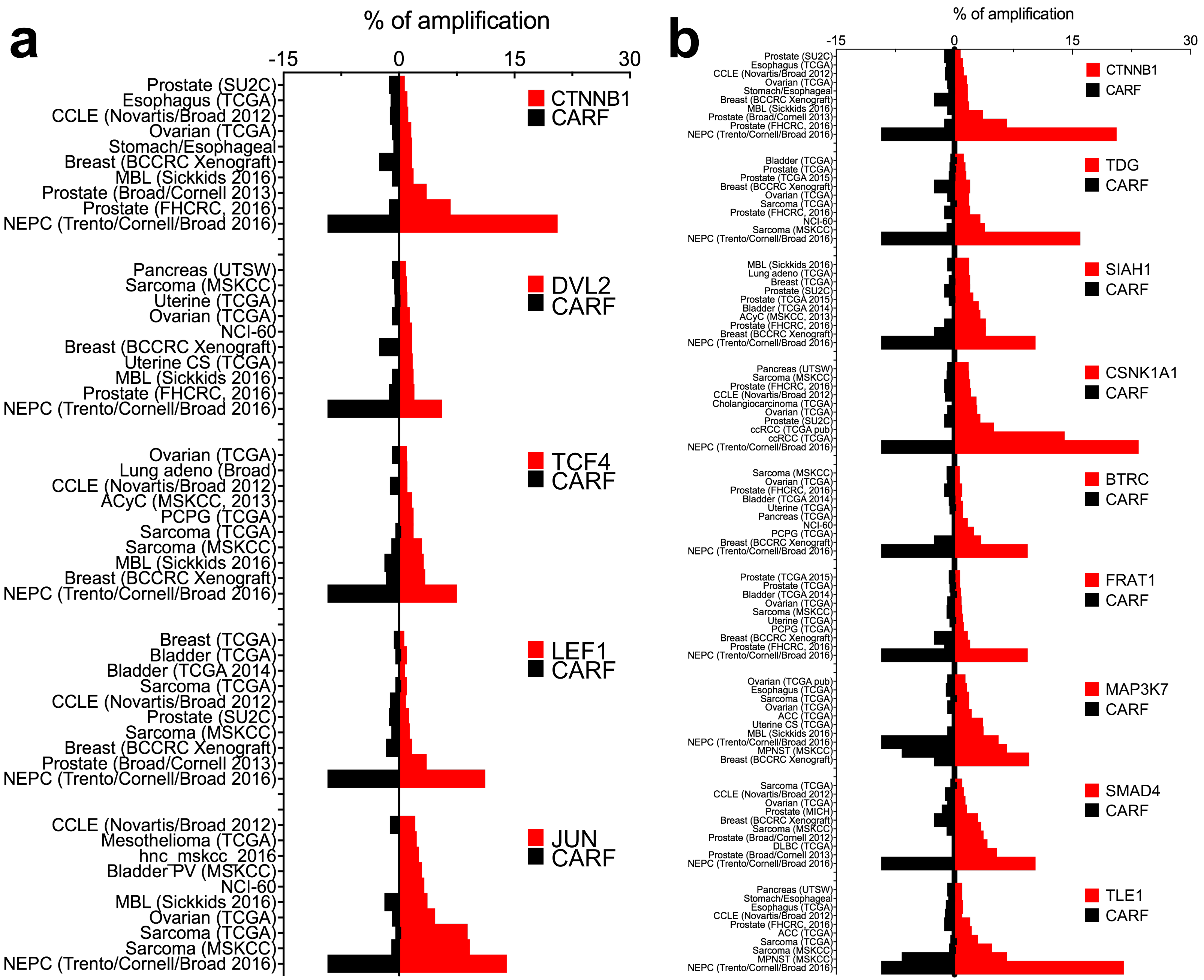
a
% of amplification
-15
0
15
30
Prostate (SU2C)
Esophagus (TCGA)
CCLE (Novartis/Broad 2012)
Ovarian (TCGA)
Stomach/Esophageal
Breast (BCCRC Xenograft)
MBL (Sickkids 2016)
Prostate (Broad/Cornell 2013)
Prostate (FHCRC, 2016)
NEPC (Trento/Cornell/Broad 2016)
CTNNB1
CARF
Pancreas (UTSW)
Sarcoma (MSKCC)
Uterine (TCGA)
Ovarian (TCGA)
NCI-60
Breast (BCCRC Xenograft)
Uterine CS (TCGA)
MBL (Sickkids 2016)
Prostate (FHCRC, 2016)
NEPC (Trento/Cornell/Broad 2016)
DVL2
CARF
Ovarian (TCGA)
Lung adeno (Broad)
CCLE (Novartis/Broad 2012)
ACyC (MSKCC, 2013)
PCPG (TCGA)
Sarcoma (TCGA)
Sarcoma (MSKCC)
MBL (Sickkids 2016)
Breast (BCCRC Xenograft)
NEPC (Trento/Cornell/Broad 2016)
TCF4
CARF
Breast (TCGA)
Bladder (TCGA)
Bladder (TCGA 2014)
Sarcoma (TCGA)
CCLE (Novartis/Broad 2012)
Prostate (SU2C)
Sarcoma (MSKCC)
Breast (BCCRC Xenograft)
Prostate (Broad/Cornell 2013)
NEPC (Trento/Cornell/Broad 2016)
LEF1
CARF
CCLE (Novartis/Broad 2012)
Mesothelioma (TCGA)
hnc_mskcc_2016
Bladder PV (MSKCC)
NCI-60
MBL (Sickkids 2016)
Ovarian (TCGA)
Sarcoma (TCGA)
Sarcoma (MSKCC)
NEPC (Trento/Cornell/Broad 2016)
JUN
CARF
b
% of amplification
-15
0
15
30
Prostate (SU2C)
Esophagus (TCGA)
CCLE (Novartis/Broad 2012)
Ovarian (TCGA)
Stomach/Esophageal
Breast (BCCRC Xenograft)
MBL (Sickkids 2016)
Prostate (Broad/Cornell 2013)
Prostate (FHCRC, 2016)
NEPC (Trento/Cornell/Broad 2016)
CTNNB1
CARF
Bladder (TCGA)
Prostate (TCGA)
Prostate (TCGA 2015)
Breast (BCCRC Xenograft)
Ovarian (TCGA)
Sarcoma (TCGA)
Prostate (FHCRC, 2016)
NCI-60
Sarcoma (MSKCC)
NEPC (Trento/Cornell/Broad 2016)
TDG
CARF
MBL (Sickkids 2016)
Lung adeno (TCGA)
Breast (TCGA)
Prostate (SU2C)
Prostate (TCGA 2015)
Bladder (TCGA 2014)
ACyC (MSKCC, 2013)
Prostate (FHCRC, 2016)
Breast (BCCRC Xenograft)
NEPC (Trento/Cornell/Broad 2016)
SIAH1
CARF
Pancreas (UTSW)
Sarcoma (MSKCC)
Prostate (FHCRC, 2016)
CCLE (Novartis/Broad 2012)
Cholangiocarcinoma (TCGA)
Ovarian (TCGA)
Prostate (SU2C)
ccRCC (TCGA pub)
ccRCC (TCGA)
NEPC (Trento/Cornell/Broad 2016)
CSNK1A1
CARF
Sarcoma (MSKCC)
Ovarian (TCGA)
Prostate (FHCRC, 2016)
Bladder (TCGA 2014)
Uterine (TCGA)
Pancreas (TCGA)
NCI-60
PCPG (TCGA)
Breast (BCCRC Xenograft)
NEPC (Trento/Cornell/Broad 2016)
BTRC
CARF
Prostate (TCGA 2015)
Prostate (TCGA)
Bladder (TCGA 2014)
Ovarian (TCGA)
Sarcoma (MSKCC)
Uterine (TCGA)
PCPG (TCGA)
Breast (BCCRC Xenograft)
Prostate (FHCRC, 2016)
NEPC (Trento/Cornell/Broad 2016)
FRAT1
CARF
Ovarian (TCGA pub)
Esophagus (TCGA)
Sarcoma (TCGA)
Ovarian (TCGA)
ACC (TCGA)
Uterine CS (TCGA)
MBL (Sickkids 2016)
NEPC (Trento/Cornell/Broad 2016)
MPNST (MSKCC)
Breast (BCCRC Xenograft)
MAP3K7
CARF
Sarcoma (TCGA)
CCLE (Novartis/Broad 2012)
Ovarian (TCGA)
Prostate (MICH)
Breast (BCCRC Xenograft)
Sarcoma (MSKCC)
Prostate (Broad/Cornell 2012)
DLBC (TCGA)
Prostate (Broad/Cornell 2013)
NEPC (Trento/Cornell/Broad 2016)
SMAD4
CARF
Pancreas (UTSW)
Stomach/Esophageal
Esophagus (TCGA)
CCLE (Novartis/Broad 2012)
Prostate (FHCRC, 2016)
ACC (TCGA)
Sarcoma (TCGA)
Sarcoma (MSKCC)
MPNST (MSKCC)
NEPC (Trento/Cornell/Broad 2016)
TLE1
CARF

Sup Fig. 6

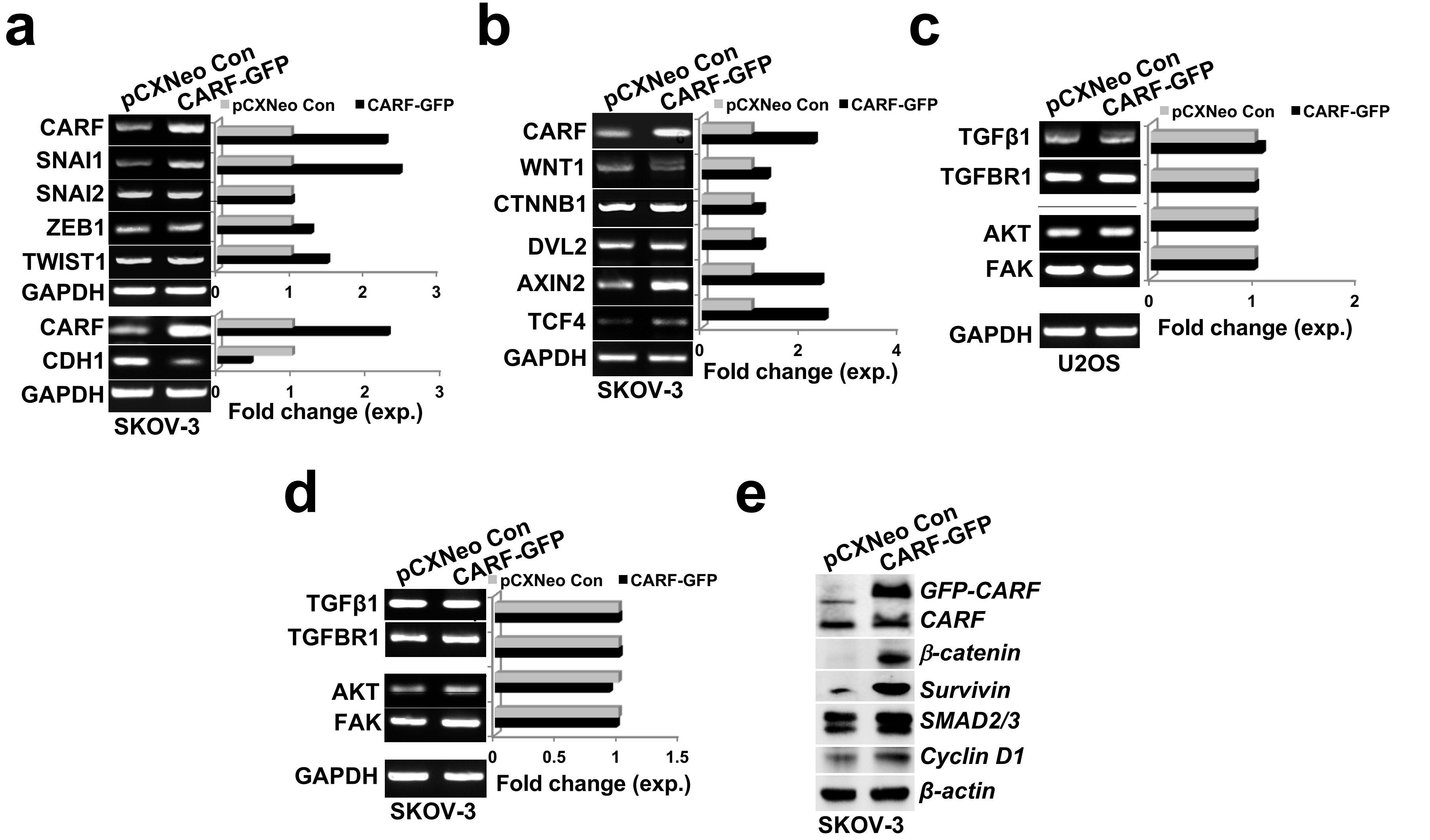

a
pCXNeo Con
CARF-GFP
pCXNeo Con
CARF-GFP
CARF
SNAI1
SNAI2
ZEB1
TWIST1
GAPDH
CARF
CDH1
GAPDH
0
1
2
3
Fold change (exp.)
SKOV-3
b
pCXNeo Con
CARF-GFP
CARF
WNT1
CTNNB1
DVL2
AXIN2
TCF4
GAPDH
0
2
4
Fold change (exp.)
SKOV-3
c
pCXNeo Con
CARF-GFP
TGFβ1
TGFBR1
AKT
FAK
GAPDH
0
1
2
Fold change (exp.)
U2OS
d
pCXNeo Con
CARF-GFP
TGFβ1
TGFBR1
AKT
FAK
GAPDH
0
0.5
1
1.5
Fold change (exp.)
SKOV-3
e
pCXNeo Con
CARF-GFP
GFP-CARF
CARF
β-catenin
Survivin
SMAD2/3
Cyclin D1
β-actin
SKOV-3

Sup Fig. 7

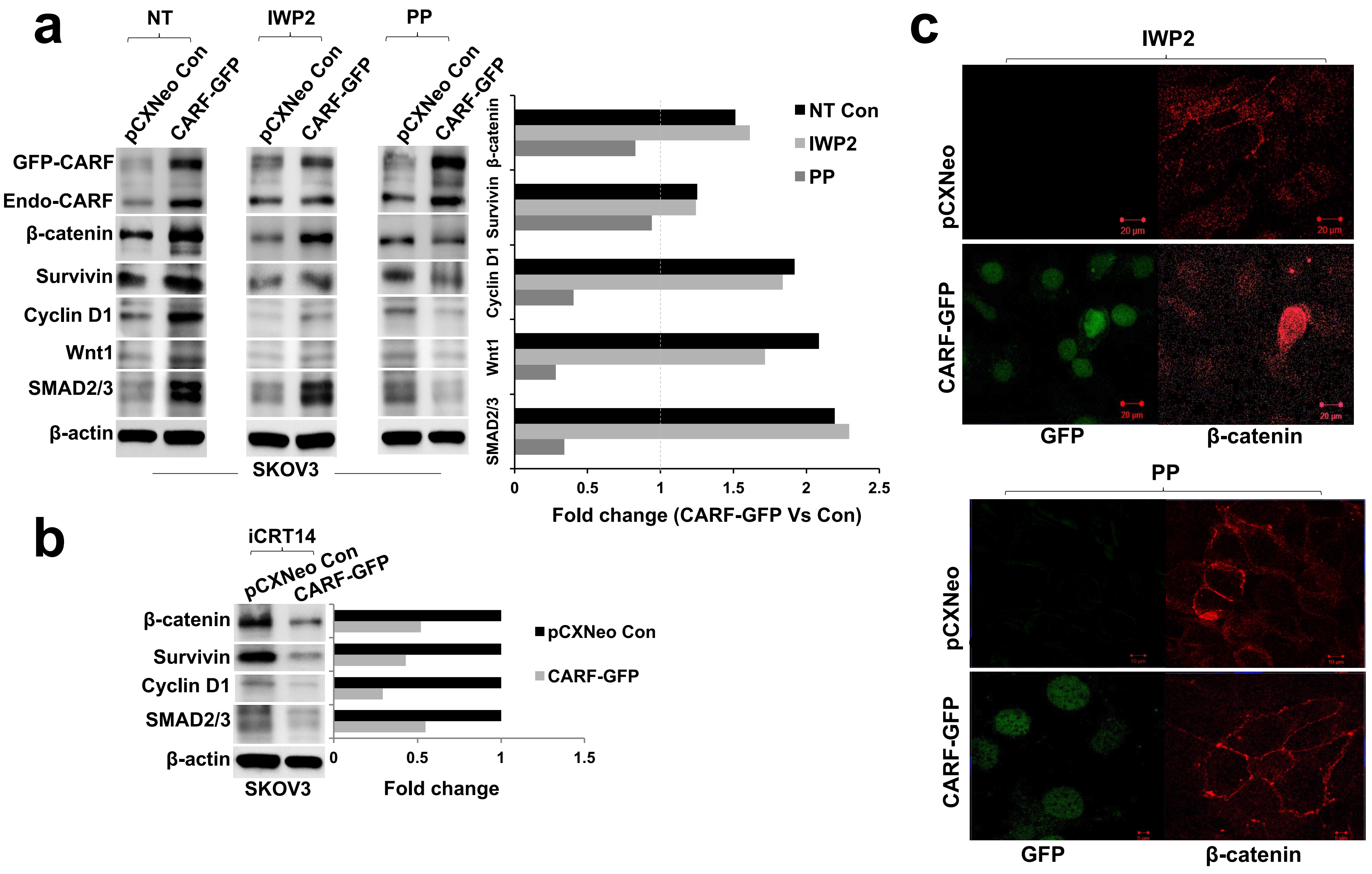

a
NT
IWP2
PP
pCXNeo Con
CARF-GFP
GFP-CARF
Endo-CARF
β-catenin
Survivin
Cyclin D1
Wnt1
SMAD2/3
β-actin
SKOV3
NT Con
IWP2
PP
0
0.5
1
1.5
2
2.5
Fold change (CARF-GFP Vs Con)
b
iCRT14
pCXNeo Con
CARF-GFP
β-catenin
Survivin
Cyclin D1
SMAD2/3
β-actin
SKOV3
0
0.5
1
1.5
Fold change
c
IWP2
pCXNeo
CARF-GFP
20 μm
GFP
β-catenin
PP
pCXNeo
CARF-GFP
GFP
β-catenin

Sup Fig. 8

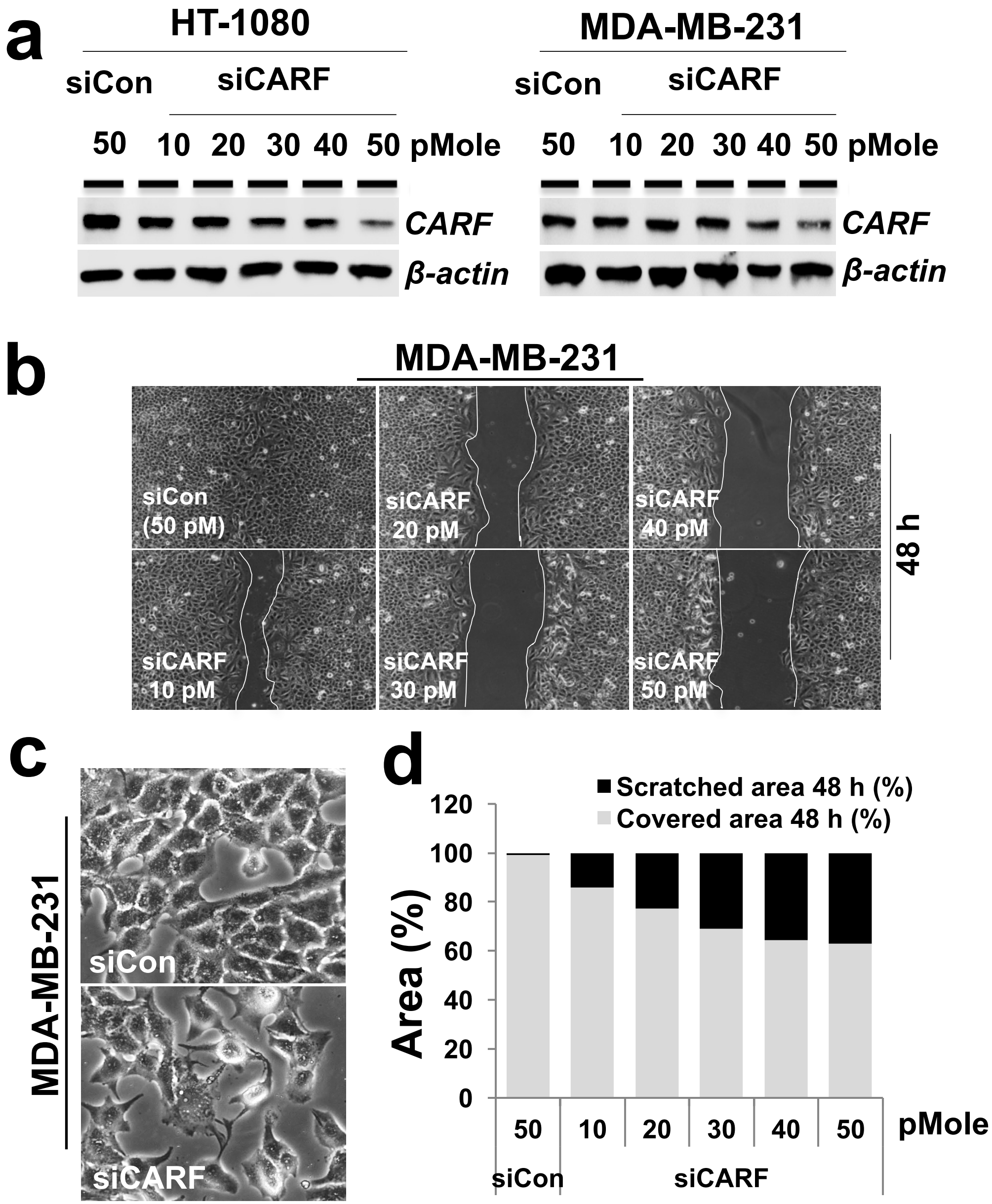

a
HT-1080
siCon
siCARF
50 10 20 30 40 50 pMole
CARF
β-actin
MDA-MB-231
siCon
siCARF
50 10 20 30 40 50 pMole
CARF
β-actin
b
MDA-MB-231
siCon (50 pM)
siCARF 20 pM
siCARF 40 pM
siCARF 10 pM
siCARF 30 pM
siCARF 50 pM
48 h
c
MDA-MB-231
siCon
siCARF
d
Scratched area 48 h (%)
Covered area 48 h (%)
Area (%)
0
20
40
60
80
100
120
50 10 20 30 40 50 pMole
siCon
siCARF

Sup Fig. 9

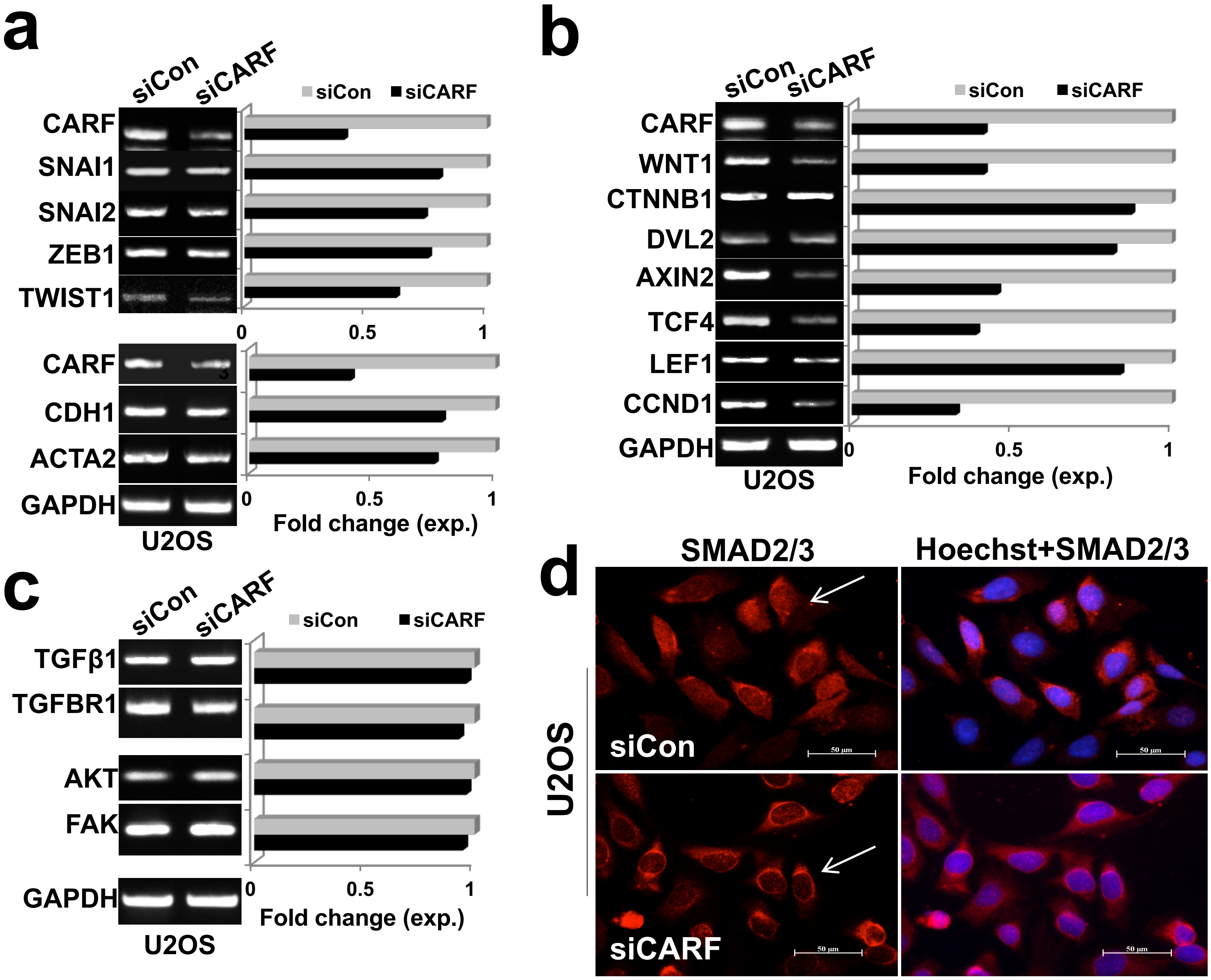
a
siCon
siCARF
siCon
siCARF
CARF
SNAI1
SNAI2
ZEB1
TWIST1
0
0.5
1
CARF
CDH1
ACTA2
GAPDH
0
0.5
1
U2OS
Fold change (exp.)
b
siCon
siCARF
siCon
siCARF
CARF
WNT1
CTNNB1
DVL2
AXIN2
TCF4
LEF1
CCND1
GAPDH
0
0.5
1
U2OS
Fold change (exp.)
c
siCon
siCARF
siCon
siCARF
TGFβ1
TGFBR1
AKT
FAK
GAPDH
0
0.5
1
U2OS
Fold change (exp.)
d
SMAD2/3
Hoechst+SMAD2/3
U2OS
siCon
siCARF
